# Nuclear dualism without extensive DNA elimination in the ciliate *Loxodes magnus*

**DOI:** 10.1073/pnas.2400503121

**Published:** 2024-09-19

**Authors:** Brandon K. B. Seah, Aditi Singh, David E. Vetter, Christiane Emmerich, Moritz Peters, Volker Soltys, Bruno Huettel, Estienne C. Swart

**Affiliations:** ^a^Max Planck Institute for Biology, Tübingen 72076, Germany; ^b^Thünen Institute for Biodiversity, Braunschweig 38116, Germany; ^c^Faculty of Science, Eberhard Karls Universität Tübingen, Tübingen 72076, Germany; ^d^Friedrich Miescher Laboratory, Tübingen 72076, Germany; ^e^Max Planck Genome Centre Cologne, Max Planck Institute for Plant Breeding Research, Cologne 50829, Germany

**Keywords:** genome editing, macronucleus, micronucleus, Ciliophora, mobile elements

## Abstract

Most eukaryotes have one nucleus and nuclear genome per cell. Ciliates have instead evolved distinct nuclei that coexist in each cell: a silent germline vs. transcriptionally active somatic nuclei. In the best-studied model species, both nuclei can divide asexually, but only germline nuclei undergo meiosis and karyogamy during sex. Thereafter, thousands of DNA segments, called internally eliminated sequences (IESs), are excised from copies of the germline genomes to produce the streamlined somatic genome. In *Loxodes*, however, somatic nuclei cannot divide but instead develop from germline copies even during asexual cell division, which would incur a huge overhead cost if genome editing was required. Here, we purified and sequenced both genomes in *Loxodes magnus* to see whether their nondividing somatic nuclei are associated with differences in genome architecture. Unlike in other ciliates studied to date, we did not find canonical germline-limited IESs, implying *Loxodes* does not extensively edit its genomes. Instead, both genomes appear large and equivalent, replete with retrotransposons and repetitive sequences, unlike the compact, gene-rich somatic genomes of other ciliates. Two other hallmarks of nuclear development in ciliates—domesticated DDE-family transposases and editing-associated small RNAs—were also not found. Thus, among the ciliates, *Loxodes* genomes most resemble those of conventional eukaryotes. Nonetheless, base modifications, histone marks, and nucleosome positioning of vegetative *Loxodes* nuclei are consistent with functional differentiation between actively transcribed somatic vs. inactive germline nuclei. Given their phylogenetic position, it is likely that editing was present in the ancestral ciliate but secondarily lost in the *Loxodes* lineage.

## Significance

Developmental genome editing exemplifies the evolution of biological complexity: Why do some eukaryotes carry extra genetic material that is excised in a complicated, costly, and time-consuming manner following sex? Ciliates are among the best-studied models of this phenomenon, however, this study reports a species where extensive editing could not be detected, but which nonetheless maintains substantial differences in DNA modifications and chromatin between its actively transcribed somatic nuclei and silent germline nuclei. This demonstrates that extensive genome editing is not a prerequisite for ciliate nuclear functional differentiation, and challenges the conventional theories about editing: that it is necessary as defense against mobile elements, and that editing, once gained, cannot be lost because of an evolutionary ratchet.

Unlike most eukaryotes, ciliates maintain two types of nuclei per cell: smaller, silent germline micronuclei (MICs) and larger, transcribed somatic macronuclei (MACs) (nuclear dualism). Reflecting their transcriptional differences, the two ciliate nuclei differ in chromatin organization and DNA modifications: MICs and MACs use different histone variants (1) and have different patterns of histone marks (2, 3). Nucleosomes are distinctly phased relative to gene features in MACs but not MICs (4), and >1% of adenosines in MAC DNA are modified as N6-methyl-deoxyadenosine (6mA), compared to negligible levels in MICs (5–8).

Only MICs leave sexual progeny (hence “germline”), while MACs are evolutionary dead ends that themselves develop from MICs, during which their genomes undergo profound, irreversible genetic changes in sequence content and organization. In most ciliates, both nuclei can divide asexually, but during sex, only MICs undergo meiosis and karyogamy to form a diploid zygotic nucleus. At least one daughter nucleus remains as a MIC, while others develop (via an intermediate, “MAC anlagen”) into new MACs to replace the old MACs (9). During development, much of the MIC genome is eliminated (10–12); this is known as germline-limited sequence and is largely composed of repetitive elements. Known MIC genomes are hence ~10 to 450 Mbp larger than MACs (~40 to 100 Mbp) (13–21). The remaining DNA (macronuclear-destined sequence) is amplified, producing 10 to 10,000 s of copies depending on species (2, 9, 10, 22), to form mature “ampliploid” MAC (11). Breakage of MIC chromosomes generates shorter MAC DNA molecules (9), with an extreme of kilobase-sized single-gene “nanochromosomes,” e.g., in spirotrichs (21, 23).

In most cases, flanking segments are joined after excision of germline-limited sequences; the latter are hence called “internally eliminated sequences” (IESs). IES length, placement, and content are variable (2, 9), e.g., mostly <100 bp in *Paramecium* but ~10 kbp in *Tetrahymena* (14, 15). The excisases that remove IESs have evolved from DNA transposases, and many IESs are transposon derivatives (15–17, 24–27). Excision is thought to be guided by development-specific small RNAs, using the old MAC as a template (28–32). Genome editing removes most mobile elements from the MAC, as a result, they are rarely exposed to natural selection and tend to accumulate in MIC genomes over time (16, 27, 33). Some other eukaryotes edit their genomes (34), but ciliates are the largest known clade where editing is pervasive. Moreover, as unicellular organisms, ciliates must maintain both the edited somatic and unedited germline genomes within the same cell.

**Fig. 1.**
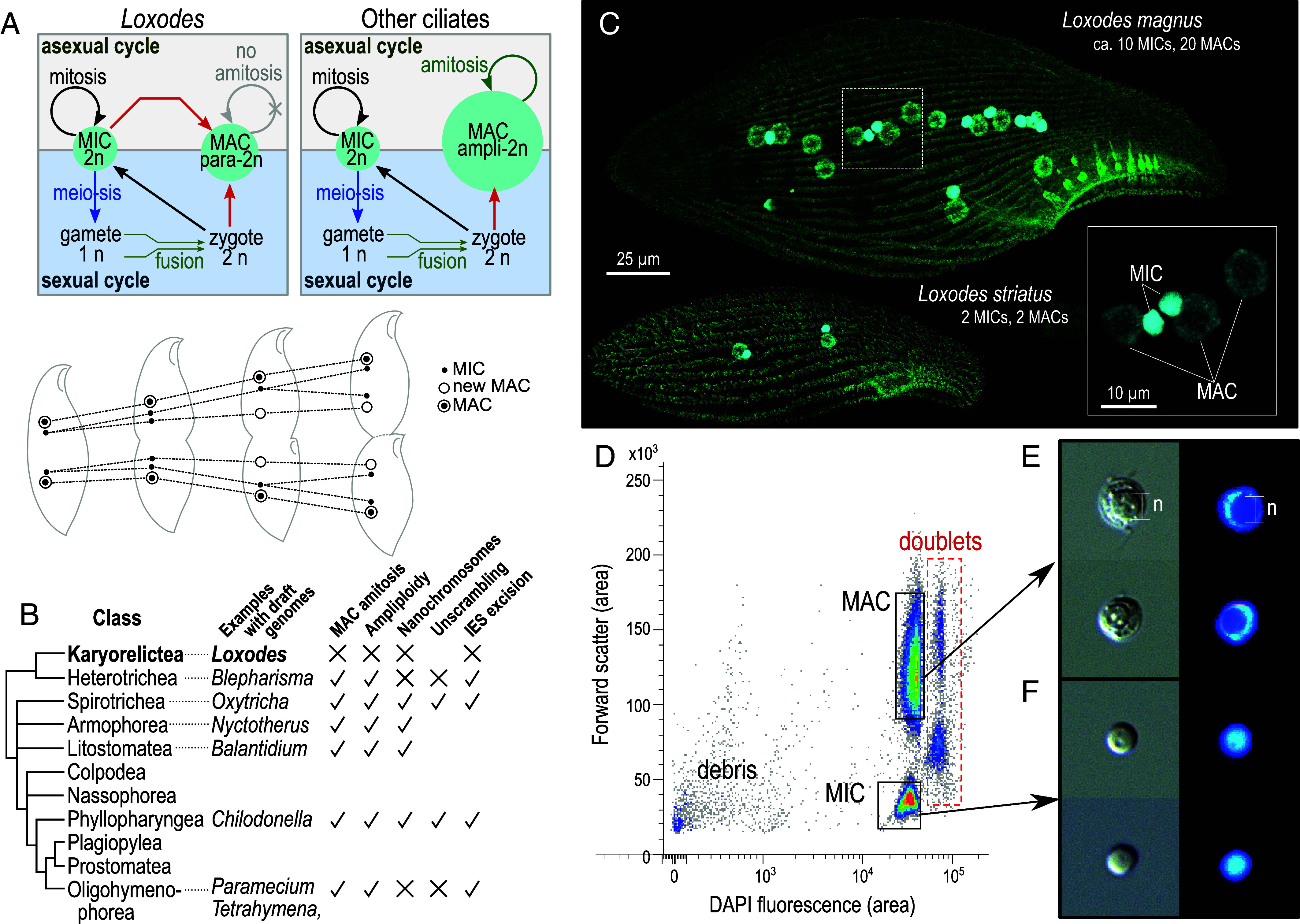
*Loxodes* nuclei purification. (*A*) Simplified diagram of nucleus development in typical ciliates vs. *Loxodes* and other karyorelicts (above); nuclei in *L. striatus* during asexual division, after (35) (below). (*B*) Diagrammatic tree of ciliate classes [after (37), branch lengths arbitrary] and genome architecture characteristics with evidence from draft genomes. (*C*) Confocal scanning fluorescence micrographs of *Loxodes* cells (maximum-intensity projections): green, alpha-tubulin secondary immunofluorescence; cyan, DAPI staining of nuclei; *Inset*, detail of *L. magnus* nuclei. (*D*) Representative flow cytometric scatterplot of forward scatter vs. DAPI fluorescence for *L. magnus* cell lysate (39,312 events depicted), with gates for MAC and MIC defined for flow sorting. Median integrated DAPI fluorescence for MACs was 116% that of MICs. (*E* and *F*) MAC and MIC respectively after sorting, imaged with differential interference contrast (*Left*) and DAPI fluorescence (*Right*); each subpanel width 10 µm. The spherical nucleolus (“n”) is less densely stained (panel *E*).

The exceptional class Karyorelictea differs from other ciliates because i) their MACs cannot divide and always develop from MIC precursors, even during asexual division (35), and ii) karyorelict MACs are less amplified than other ciliates (“paradiploid” vs. ampliploid); e.g., in *Loxodes magnus* the DNA content in MACs is only up to twice that of MICs (36) (Fig. 1 *A* and *B*). Like other ciliates, only MICs have been observed to participate in meiotic sex in *Loxodes*, while their MACs have prominent nucleoli and active RNA synthesis (36), consistent with germline and somatic roles respectively. What consequences does decoupling MIC-to-MAC development from sex have for genome development and evolution? If they have IESs, these would need to be excised at every cell division, not just after sex.

We can now put karyorelict genome architecture in evolutionary context, as we have recently characterized genome editing in a representative of Heterotrichea (17, 20), the sister group to the karyorelicts (37, 38). Heterotrichs have dividing, ampliploid MACs like other ciliates, so nondividing paradiploid MACs in karyorelicts must be a derived character. We therefore purified MICs and MACs from two *Loxodes* species (Fig. 1*C*) to compare their genomes, examining *L. magnus* in more detail. Unexpectedly we did not detect classical IESs, although their nuclei are distinct in terms of chromatin organization and DNA methylation, suggesting that their genomes are on a different evolutionary trajectory from other ciliates studied to date.

## Results

### Physical Purification of *Loxodes* MICs and MACs.

Two distinct clusters corresponding to MACs and MICs were observed in fluorescence activated nuclear sorting of DAPI-stained cell lysates of *L. magnus* (Fig. 1 and *SI Appendix*, Fig. S1*A*) and *Loxodes striatus* (*SI Appendix*, Fig. S1*B*). MACs had higher forward scatter and DAPI fluorescence than MICs (Fig. 1*D*), and their identities were confirmed with microscopy after sorting by presence of nucleoli (Fig. 1 *E* and *F*). Sorted nuclear purity was also verified by known chromatin and DNA-modification differences between ciliate MICs and MACs: histone marks, nucleosome positioning, and 6mA base modifications (*Results*: “*Loxodes MACs Have Characteristics of Both Active Chromatin and Heterochromatin*”).

### *Loxodes* MIC and MAC Genome Libraries Have Similar k-mer Composition.

**Fig. 2.**
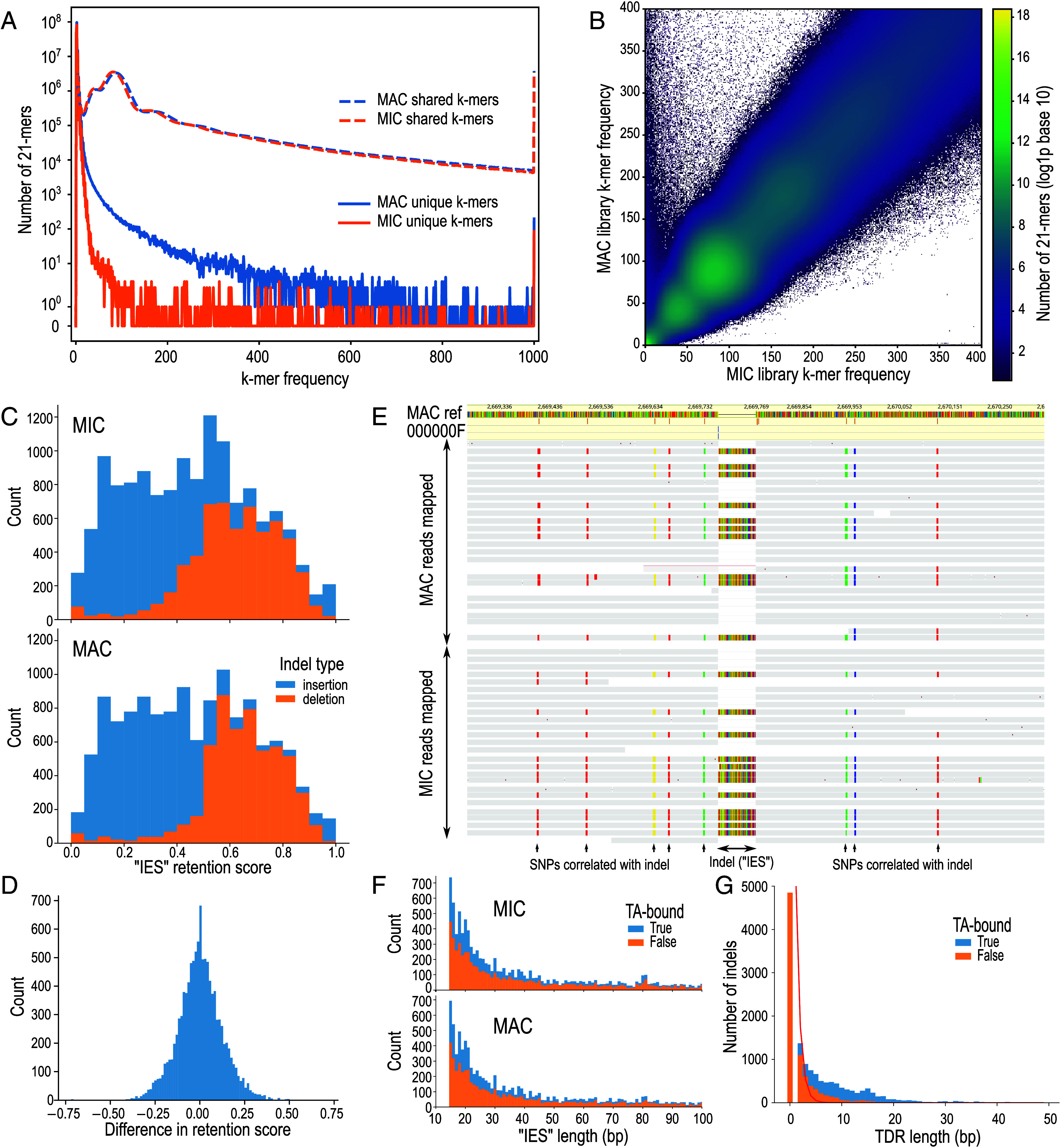
Screening for IESs in *L. magnus*. (*A*) k-mer multiplicity plot for shared (dashed lines) vs. unique (solid lines) 21-mers in MAC (blue) and MIC (orange) sequence libraries. (*B*) Heatmap comparing frequency of genomic 21-mers in MIC vs. MAC; color scale represents log (1+number of k-mers); axes truncated at 400× frequency. (*C*) Histograms of relative coverage (“retention score”) for putative “IESs” (indels) predicted by an IES detection pipeline from MIC and MAC HiFi long-read libraries. (*D*) Histogram of differences in retention scores between MIC and MAC libraries for putative “IESs.” (*E*) Example of HiFi long reads (horizontal bars) from MIC and MAC mapped to MAC reference genome (colored bar, *Top*), containing an “IES” indel correlated with SNPs; colored bases in reads differ from reference. (*F*) Length histograms of indel polymorphisms; colored by whether they are bound by TA-containing tandem direct repeats (TDRs); *x*-axis truncated at 100 bp. (*G*) Lengths of TDRs bounding indel polymorphisms (bars), compared to expected lengths assuming random sequence (red line).

We first compared the composition of short subsequences of defined length, known as k-mers, in unassembled short-read *Loxodes* MIC and MAC genome libraries (k = 21 nt). Most k-mers observed were shared by both libraries. Of k-mers with combined frequency ≥5× in *L. magnus*, only 3.3% were unique to one or the other library, whereas 93% were observed ≥5× in each library. Unique k-mers lacked discernible frequency peaks (Fig. 2*A*), nor was there an obvious cluster of k-mers with different coverage between the two libraries (Fig. 2*B*), contrary to what would be expected if much of the genome was germline-limited like in other ciliates (*SI Appendix*, *SI Results 1*), or if specific loci were differentially amplified, as previously proposed (36, 39). There was no evidence for amplification of rRNA in particular (*SI Appendix*, *SI Results 2*).

k-mer frequency spectra in each nucleus showed a main coverage peak (~85×), a heterozygosity peak (~40×), and additional peaks (~170× and ~430×) that suggested some degree of genome duplication or paralogy. The spectra were long-tailed; 0.68% of k-mers had ≥1,000× frequency, representing high-copy-number repeat elements. Low-frequency k-mers were likely sequencing errors (18% singletons, 56% with combined frequency <5×) (Fig. 2*A*). Model-fitting of k-mer coverage spectra peaks predicted similar genome sizes (262 Mbp MIC, 261 Mbp MAC) and heterozygosity (0.60% and 0.59%), although these do not account for high copy repeats. *L. striatus* k-mer spectra showed similar patterns (*SI Appendix*, Figs. S3 and S4).

### Classical IESs Not Detected in *L. magnus* MIC by Mapping to MAC Reference Genome.

We next attempted to detect IESs in *L. magnus* by mapping error-corrected long reads (PacBio HiFi) from sorted MICs and MACs to the MAC reference assembly. Any MIC-specific IESs should appear in mapped reads as insertions relative to the MAC reference, and the fraction of reads bearing the insert (“retention score”) should be significantly higher in reads from the MIC than MAC; this approach has been used to detect and assemble IESs in other ciliates (40–42).

Although slightly more candidate “IESs” were called from the *L. magnus* MIC genome than the MAC genome (13,734 vs. 12,897 respectively, 10,992 in both), the mean retention scores per library were similar (0.45 for MIC, 0.46 for MAC) (Fig. 2*C*), and retention scores of shared “IESs” were not significantly different between the two libraries (Fig. 2*D*, Wilcoxon signed-rank test, one-sided for higher score in MIC, *P* = 0.29). The ratio of insertions vs. deletions was similar between MIC and MAC libraries (Fig. 2*C*), contrary to the expectation of more inserts in the MIC library. Inserts that were both unique to the MIC library and with high retention score (>0.9), as expected of true IESs, were few in number (forty) and located in regions of low coverage (mean 4.2×), and were hence probably mispredictions due to insufficient coverage.

“IESs” from *L. magnus* were instead consistent with monoallelic indels or mobile element insertions at heterozygous loci (*SI Appendix*, *SI Results 3*). The insertions (putative “IESs”) had a coverage of about 50%, consistent with the genome’s diploid ploidy inferred from coverage of single nucleotide polymorphisms (SNPs), and an insert’s presence in a given read was usually correlated with the SNP-based haplotype of that read, instead of the nucleus type (Fig. 2*E* and *SI Appendix*, Fig. S5 and *SI Results 3*). We do not expect true IESs to be so closely correlated with haplotypes but rather to be fixed in the population. The insertion length distribution sloped downward from the 15 bp lower length cutoff (Fig. 2*F*), unlike IES length distributions of other ciliates, which are longer (e.g., min. 26 bp in *Paramecium*), and with peaks at specific lengths. More insertions were bound by terminal direct repeats (TDR) than expected by chance, especially TDRs that contain TA-sequence submotifs (Fig. 2*G*), so many insertions could originate from mobile elements (see below).

### Both *L. magnus* Nuclear Genomes Are Rich in Tandem and Interspersed Repeats.

*L. magnus* genome assemblies from long reads were large (MIC 848 Mbp, MAC 706 Mbp), but a large fraction comprised low-complexity tandem repeats (MIC 359 Mbp, MAC 231 Mbp) (*SI Appendix*, Fig. S6). About one million interspersed repeats from 915 families were annotated per assembly, covering 571 Mbp (MIC) and 454 Mbp (MAC), mostly unclassified (757 k copies, 366 Mbp total length in MAC). Interspersed repeat and low-complexity tandem repeat annotations overlapped substantially. Genome sizes after repeat masking were similar (MIC 245 Mbp, MAC 229 Mbp) and closer to initial k-mer-based size predictions (*SI Appendix*, Table S1). The difference in total assembly sizes is likely caused by misassembly of low-complexity repeats, rather than by imprecise elimination of repetitive elements in addition to precise IES excision, as has been found in the ciliate *Paramecium* (43), because unassembled reads from MICs vs. MACs have a similar proportion of low complexity sequences (Fig. 2*A* and *SI Appendix*, Fig. S7).

Among the repeats were hundreds of ribosomal RNA genes (rDNA) at comparable numbers in both genomes (*SI Appendix*, *SI Results 2*). *L. magnus* rDNAs appear to be chromosomal sequences organized in head-to-tail tandem arrays, the common eukaryotic manner, instead of amplified extrachromosomal DNA molecules characteristic of *Tetrahymena*, *Paramecium,* and *Oxytricha*.

### Gene Prediction for Context-Dependent Sense/Stop Codons.

Karyorelicts, including *Loxodes*, use an ambiguous stop/sense genetic code (NCBI translation table 27) where the only stop codon, UGA, can also encode tryptophan (W) if sufficiently far upstream of the mRNA poly-A tail (44, 45). Coding UGAs must be distinguished from stop UGAs to predict genes, but existing software does not permit single codons to have alternative, context-dependent translation outcomes.

**Fig. 3.**
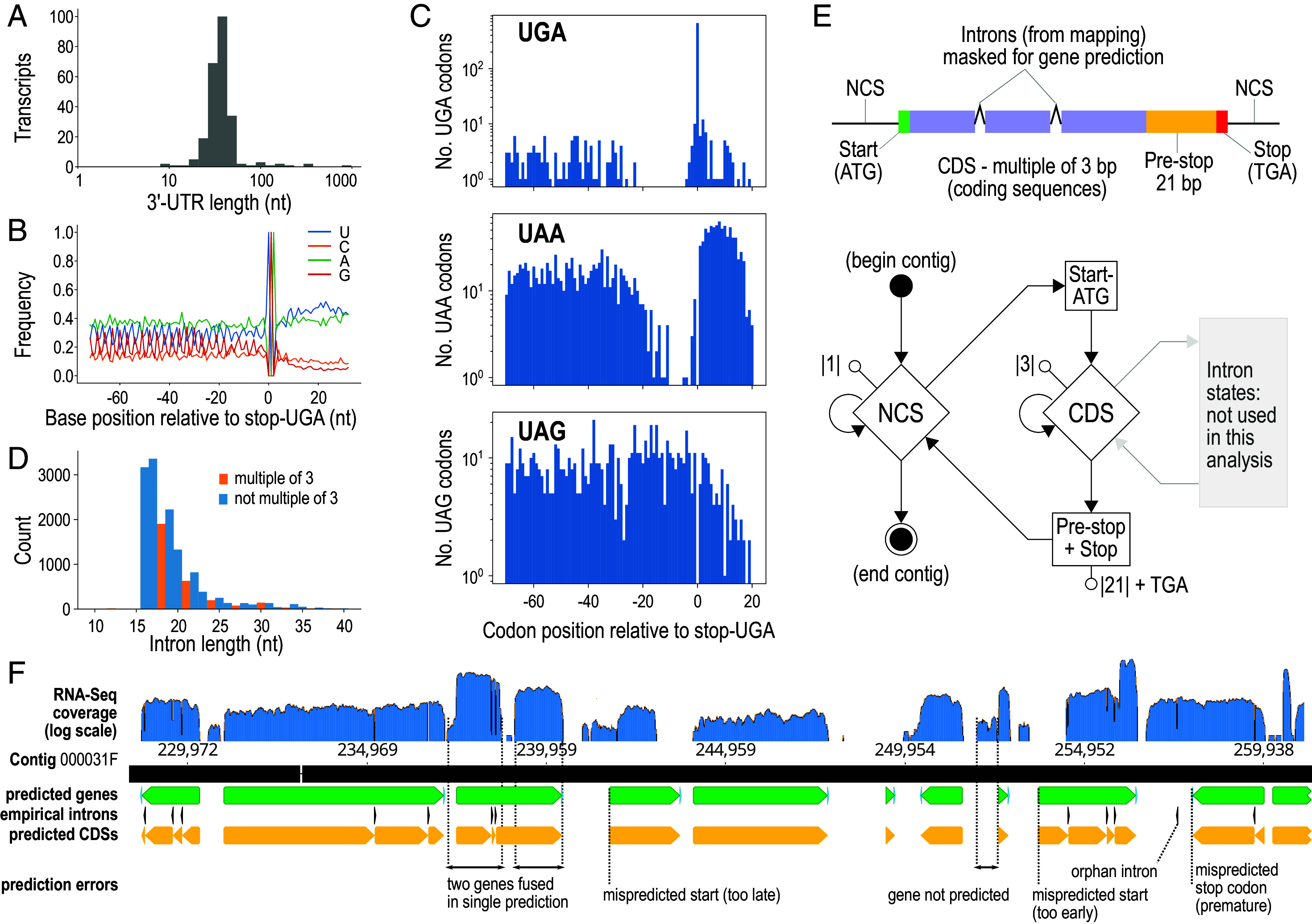
*L. magnus* gene prediction. (*A*) Length distribution of 3′-untranslated regions (3′-UTRs) from poly-A-tailed transcripts with stop codons predicted from BLASTX hits to other ciliates. (*B*) Base composition around predicted stop codons in transcripts. (*C*) Counts of UGA, UAA, and UAG codons relative to predicted stop-UGA codons in transcripts, showing depletion of in-frame UGA and UAA immediately upstream of stop-UGAs, but no depletion of UAG (cf. *SI Appendix*, Fig. S7). (*D*) Length distribution of introns predicted from RNA-seq mapping to MAC assembly (excluding orphan introns). (*E*) Diagrams of gene model and GHMM used for gene prediction. Start, CDS, and stop states in the GHMM are also mirrored by their corresponding reverse complements. Introns were annotated empirically from RNA-seq mapping. (*F*) Excerpt of Pogigwasc gene prediction from MAC contig 000031F; annotation tracks for predicted genes (green), CDSs (yellow), empirical introns (black), aligned against RNA-seq coverage (blue). Common types of mispredictions recognizable by comparison with RNA-seq mappings are indicated.

Assembled transcripts with poly-A tails ≥7 bp and with BLASTX hits to published ciliate proteins revealed informative sequence characteristics for predicting stop UGAs in *L. magnus*. Like other ciliates, their 3′-untranslated regions (3′-UTRs) were short (mean 53 bp, median 41 bp) (Fig. 3*A*). Coding sequences (CDSs) were more GC-rich than 3′-UTRs (33.5% GC vs. 18.6% respectively), and showed a 3-base periodicity in their base composition associated with codon triplets (Fig. 3*B*). Coding UGAs and UAAs were depleted for about 20 codon positions before the putative true stop UGA (Fig. 3*C*), unlike other codons (*SI Appendix*, Fig. S8).

Introns identified by RNA-seq mapping to the MAC assembly were much shorter than in typical eukaryotes (mean 19.3 bp, mode 17 bp, 93% with length ≤25 bp; <16 bp negligible) (Fig. 3*D*), but nonetheless longer than in heterotrichs, the sister group to karyorelicts, where ~95% of introns were 15 bp and the remainder 16 bp (20, 22). Introns with lengths of a multiple of three (3*n*-introns) were relatively depleted (Fig. 3*D*), as previously observed in oligohymenophorean and spirotrich ciliates (46, 47).

We adapted an existing generalized hidden Markov model (GHMM) (48) for *L. magnus* gene prediction, adding a probabilistic state for the codon UGA (either W or Stop), and a region of 21 nt before stop-UGAs wherein no in-frame UGAs are permitted, to represent the observed depletion of coding UGAs immediately upstream of the stop UGA (Fig. 3*E*). *L. magnus* introns were difficult to model because of their short length and unusual length distribution, so we annotated them empirically from RNA-Seq mappings. We implemented the GHMM in our software Pogigwasc (49) and parameterized it with a set of 152 manually annotated genes (50). 94% completeness was estimated by BUSCO (Alveolata marker set) from the predicted proteome (*SI Appendix*, Fig. S9 and *SI Results 4*).

### Searches for Genes and Small RNAs Related to Genome Editing.

**Fig. 4.**
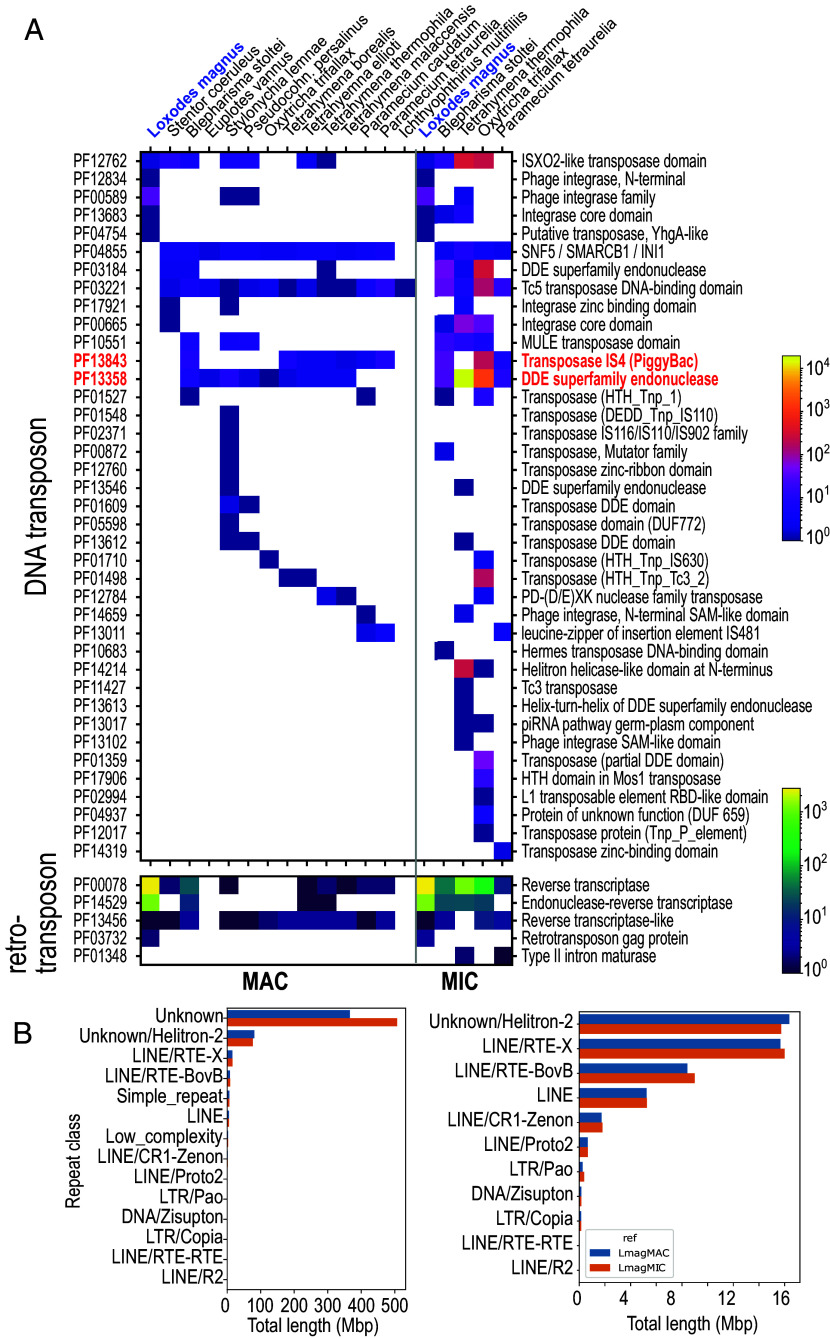
Repeats and mobile element domains in *L. magnus* compared to other ciliates. (*A*) Heatmaps representing Pfam domain counts related to mobile elements per MAC (*Left*) or MIC (*Right*) genome in ciliates. Red text: domains associated with known or proposed genome editing excisases (PF13843, PF13358). (*B*) Total lengths of interspersed repeat annotations in MIC (orange) vs. MAC (blue) genomes, sorted by classification. *Left*: All categories. *Right*: “Unknown” and rnd-1_family-2 (“Unknown/Helitron-2”) excluded to show details.

The *L. magnus* genome assembly encoded no detectable homologs of proposed domesticated ciliate IES excisases. Neither the DDE_Tnp_1_7 (Pfam PF13843) domain found in PiggyBac family homologs (PiggyMacs, Pgm) of oligohymenophoreans and heterotrichs, nor the DDE_3 (PF13358) domain from *Oxytricha* TBE element transposases were annotated in predicted proteins (Fig. 4*A*). To account for incompletely predicted genes, we performed a translated search (TBLASTN) against the genomes with model ciliate Pgm and TBE-transposase protein sequences. The best hit [*Blepharisma stoltei* Pgm (17) to the *L. magnus* MIC] had an E-value of only 0.12, compared to 10^−33^ for an alignment of *Paramecium tetraurelia* Pgm to the *B. stoltei* genome that recovered the *B. stoltei* Pgm. The weak match in *L. magnus* is hence likely spurious.

Apart from domesticated excisases, other components of the ciliate genome editing toolkit are difficult to distinguish from homologs with other functions. An exception is Dicer ribonucleases: Ciliates have two Dicer classes: canonical Dicer (Dcr) for siRNA biogenesis, and development-specific Dicer-like proteins (Dcl) that lack additional Dcr N-terminal domains, which produce precursors to sRNAs involved in genome editing (30, 51). Both Dcr and Dcl homologs were found in *L. magnus* (*SI Appendix*, Fig. S10 and *SI Results 5*).

We found no evidence for editing-associated small RNAs in *L. magnus*. Since MIC-to-MAC development is obligatory following asexual division of *Loxodes*, we reasoned that if editing-associated sRNAs were present, they should be produced in actively growing but not in starved populations. However, sRNA length distributions in actively growing and starved cells were similar (peaks 24, 25 nt), unlike other ciliates, including the heterotrich *Blepharisma*, where editing-associated sRNAs form a distinct size class and are abundant during MIC-to-MAC development. Editing-associated sRNAs should map to both DNA strands, but the *Loxodes* sRNAs observed are strand-biased and probably represent antisense, gene-silencing siRNAs (*SI Appendix*, Fig. S11 and *SI Results 6*).

### Abundant Retrotransposon-Related vs. Rare DNA Transposon-Related Elements in *L. magnus*.

Thousands of copies of retrotransposon-related domains reverse transcriptase RVT_1 (PF00078, ~2,700 copies) and endonuclease Exo_endo_phos_2 (PF14529, ~1,200 copies) were encoded in both nuclear genomes of *L. magnus*. This was ~100 times the next highest counts in ciliates in the *B. stoltei* MAC genome (20), and contrasted with the paucity of DNA transposase-related domains (Fig. 4*A*).

At least two repeat families, rnd-1_family-27 and rnd-1_family-19, appeared to represent complete long interspersed nuclear elements related to LINEs and other autonomous non-LTR retrotransposons with 5 to 6 kbp consensus length; about 10% of the ~3,000 copies detected per family were full-length with low (<10%) sequence divergence from the consensus (*SI Appendix*, Table S2). They contained coding sequences with both RVT_1 and Exo_endo_phos_2 domains typical of LINEs (52). The top BLASTp hits to GenBank’s nr database for representative *Loxodes* proteins encoding these domains were to *B. stoltei* proteins, so these elements may date to the karyorelict/heterotrich common ancestor. In total, >30,000 repeat elements per genome assembly were classified by RepeatMasker as LINEs (Fig. 4*B*), most of which were incomplete and hence likely inactive (*SI Appendix*, Table S2).

504 instances of interspersed repeats overlapped closely with monoallelic indel polymorphisms (>90% reciprocal overlap), including ten full-length copies of rnd-1_family-27 and two of rnd-1_family-19. The indels incidentally confirmed that mobile element family boundaries were correctly predicted, which is otherwise difficult for non-LTR retrotransposons because they may not be bound by conserved motifs or target site duplications (53).

Unlike the retrotransposon sequences, repeats classified as helitrons or DNA transposons lacked the expected conserved domains and were likely spurious annotations (*SI Appendix*, *SI Results 8*). Additionally, two proteins with the “ISX02-like transposase” motif (PF12762, DDE_Tnp_IS1595) were related to sequences from *Blepharisma* and *Stentor* but probably no longer involved in transposition (*SI Appendix*, *SI Results 9*), and the gene containing a YhG-like transposase domain (PF04654) was associated with a gene cluster with signs of recent horizontal gene transfer from *Rickettsia* bacteria (*SI Appendix*, *SI Results 10*).

### *Loxodes* MACs Have Characteristics of Both Active Chromatin and Heterochromatin.

**Fig. 5.**
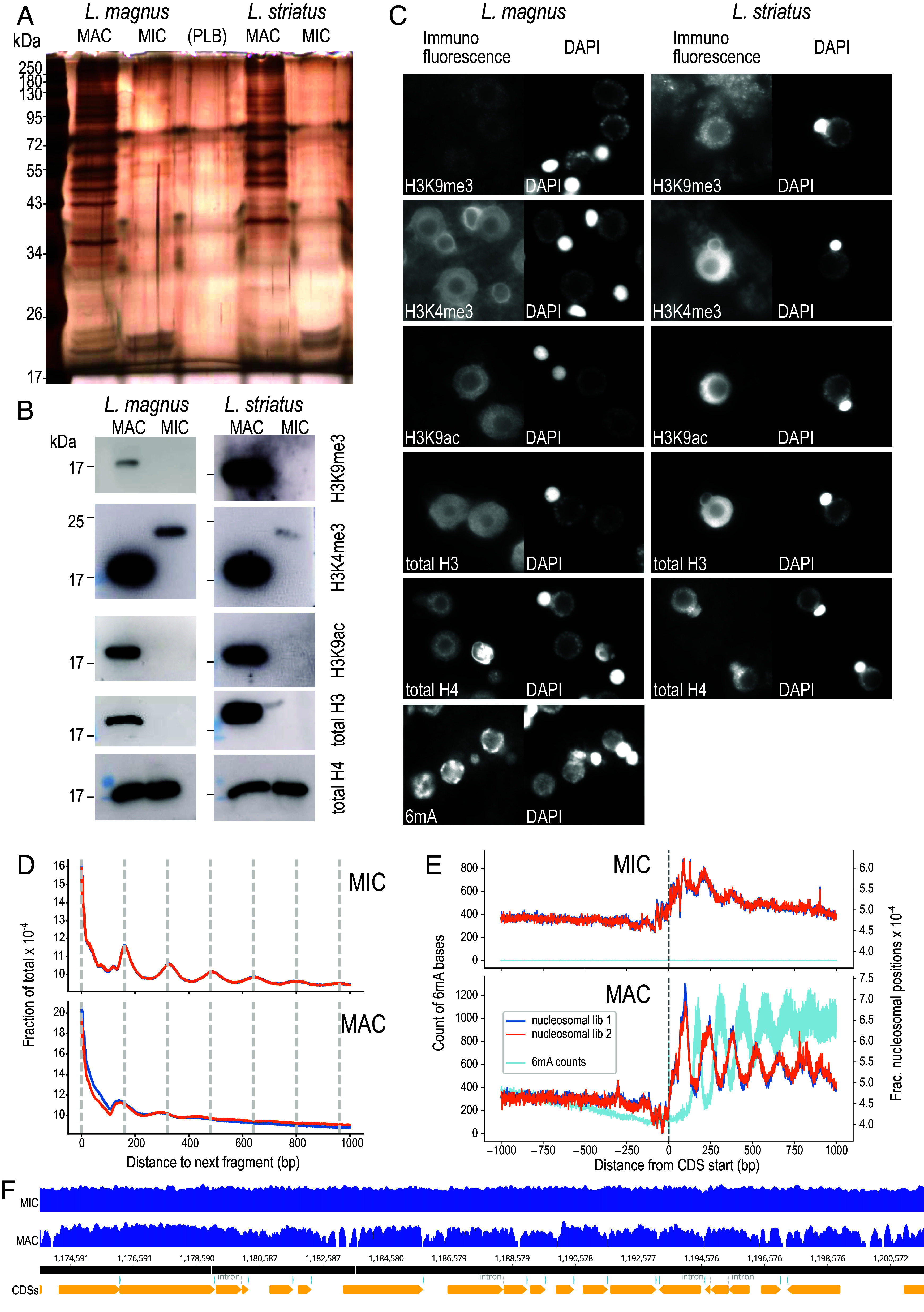
Molecular differences between *Loxodes* MICs and MACs. (*A*) Silver-stained PAGE gel of protein extracts from flow-sorted nuclei. PLB—protein loading buffer only. (*B*) Western blots against histone modifications in flow-sorted nuclei. (*C*) Secondary immunofluorescence in fixed cells against histone modifications or 6mA, alongside DAPI staining of DNA. Panel widths: 30 µm. (*D*) Global phaseograms of nucleosomal DNA density (two replicates: dark blue, orange lines) in flow-sorted *L. magnus* nuclei (low complexity repeats masked); vertical lines—160 bp intervals. (*E*) Phaseograms of nucleosomal DNA density (two replicates: dark blue, orange lines) and 6mA modified bases (light blue) relative to predicted coding sequence start positions in flow-sorted *L. magnus* nuclei. (*F*) Example coverage pileups (log scaled) for *L. magnus* MIC vs. MAC nucleosomal DNA reads mapped to MAC assembly (contig 000000F), aligned with CDS predictions (*Bottom* track).

*Loxodes* nuclei have distinct morphology (Fig. 1*C*) and chromatin organization. MAC protein composition was more diverse, as silver-stained PAGE gels revealed multiple prominent bands for MACs compared to few visible bands for MICs, of which the most prominent corresponded to typical histone sizes (Fig. 5*A*).

Histone marks typical of activation and repression were detected by western blots in MACs but not MICs (Fig. 5*B*): histone H3 lysine 9 acetylation (H3K9ac, active transcription) and H3 lysine 9 trimethylation (H3K9me3, heterochromatin). H3 lysine 4 trimethylation (H3K4me3, euchromatin) was detected in MACs at the expected size (~17 kDa) but MICs showed a weaker, higher-weight band. Immunofluorescence localization was consistent with western blots (Fig. 5*C*). As expected, histone marks in MACs were colocalized with DAPI-stained chromatin but absent from nucleoli. H3K9me3 and H3K4me3 had background signals in cytoplasm, and H3K4me3 also showed a peripheral signal surrounding MICs not colocalized with DNA. MAC localization of H3K9ac and H3K4me3 in *Loxodes* is consistent with other ciliates (54–56), whereas H3K9me3 in *Loxodes* MACs is unusual because it is usually limited to developing MACs (3, 57). H3K4me3 is limited to MACs in *Tetrahymena* (56), but in *Paramecium* was found in both MACs and MICs (55).

Total histone H3 was detected in MACs but not MICs with a commercial antibody (Fig. 5 *B* and *C*). The genome encodes multiple H3 homologs, clustering into three groups, only one of which (canonical H3-related) was likely detectable by the antibody applied (*SI Appendix*, Fig. S12 and *SI Results 11*), hence MACs probably use canonical H3 while MICs may use a different variant. Histone H4, the most conserved core histone, was detected in both nuclei (Fig. 5 *B* and *C*).

Nucleosomal positioning patterns differed between *L. magnus* MIC and MAC at both the global scale and relative to gene features. Similar dsDNase digestion conditions to isolate nucleosomal DNA yielded smaller fragments from MACs than MICs (*SI Appendix*, Fig. S13*A*). When sequenced and mapped to the genome, the global phaseogram, i.e., the distribution of nucleosomal fragment positions relative to each other, displayed periodic peaks at multiples of 160 bp, the expected length of nucleosomal plus linker DNA (Fig. 5*D*). These peaks were more pronounced in MICs than MACs, unlike in *Tetrahymena* (4). However, when phaseograms are drawn relative to the starts of predicted coding sequences, MACs display periodic peaks within coding sequences, but not MICs (Fig. 5*E*), like in *Tetrahymena* (4). Raw coverage pileups of MAC nucleosomal reads also showed arrays relative to gene features, which were not seen with MIC nucleosomal reads (Fig. 5*F*). We interpret this to mean that MIC chromatin is condensed and inactive, with nucleosomes regularly arrayed but independent of gene locations, whereas MAC chromatin is accessible, with nucleosomes arrayed relative to genes due to transcription (*SI Appendix*, Fig. S13*C*).

The base modification 6mA was abundant in *L. magnus* MACs but almost absent in MICs, according to both immunofluorescence (Fig. 5*C*) and PacBio single-molecule real-time sequencing (SMRT-Seq) [4,405,028 ApT positions (0.85%) in MAC vs. 845 (0.00013%) in MIC], consistent with other ciliates (58, 59). 99.6% of 6mA calls were in ApT motifs, which are also the exclusive motif for 6mA in *Tetrahymena* (60). 6mA coverage across *L. magnus* gene bodies was strongly biased toward the sense strand, with a ~34:1 plus to minus strand ratio (3,563,745:105,843 6mAs) in coding sequences (*SI Appendix*, Fig. S11*C*), and periodic, with an alternate phase to nucleosome positioning (Fig. 5*E*), similar to *Tetrahymena* and *Oxytricha* (6, 8). Unlike other ciliates, 6mA coverage did not fall off sharply toward the 3′ end of the gene body. *L. magnus* genes not transcribed by RNA polymerase II (e.g., rRNA) largely lacked 6mA, suggesting that 6mA methylation is coupled to RNA polymerase II transcription in the MAC, as in *Tetrahymena* (8).

Almost all ApT motifs with 6mA in *L. magnus* were hemimethylated in both MAC (99.87%) and MIC (100%) assemblies (e.g., *SI Appendix*, Fig. S11*C*), whereas other ciliate MACs have a mixture of hemi- and full methylation, including *Blepharisma* (59.4% hemi) (17), *Tetrahymena* (11% hemi) (60), and *Oxytricha* (6, 8). Full methylation is necessary for semiconservative 6mA transmission during asexual MAC division (60). Therefore, absence of full methylation in *L. magnus* is consistent with their nondividing MACs and with de novo, nonepigenetic methylation when new MACs develop. Unlike *Blepharisma*, the *L. magnus* genome lacks homologs of the entire *Tetrahymena*/*Oxytricha* 6mA methyltransferase complex. The two putative *L. magnus* DNA methyltransferase homologs resemble *Tetrahymena*’s 6mA hemimethylase, AMT2 (*SI Appendix*, *SI Results 12*).

## Discussion

Of all ciliates studied thus far, the karyorelict *L. magnus* most resembles “conventional” eukaryotes, lacking several characteristic ciliate features. To the known inability of its somatic nuclei to divide asexually, this study adds the absence of extensive IES excision, differential amplification, and somatic extrachromosomal rDNA molecules. Its somatic genome is also larger than other ciliates and replete with mobile elements.

Nonetheless, *Loxodes* maintains distinct macro- (MACs) and micronuclei (MICs). *Loxodes* MACs contain active transcription markers that are also MAC-specific in other ciliates: abundant 6mA DNA methylation, nucleosomes phased relative to genes, 6mA phased between nucleosomes, and a transcription-associated histone modification (H3K9ac). However, unlike other ciliates, *Loxodes* MAC DNA is only hemimethylated, probably reflecting their nondividing MACs, and their MACs have a heterochromatin-associated mark H3K9me3, which may suppress mobile element expression.

### Comparison with Previous Studies on Karyorelicts.

Our conclusions contradict a previous report of genome editing in an uncultivated *Loxodes* sp. (39). Their claim of up to 10^4^-fold variation in genome amplification is quantitatively unrealistic and is likely a methodological artifact, whereas their putative IESs could correspond to the indel or mobile element insertion polymorphisms described here (*SI Appendix*, *SI Discussion*). Nonetheless, other karyorelicts may have some editing: *Trachelonema sulcata* may eliminate and amplify DNA during development, as their developing MACs have distinctly less DNA than MICs or mature MACs (61). Old MACs in *Loxodes* have higher and more variable DNA content than recently matured MACs (36), which may be nonspecific amplification in senescent nuclei, as we did not observe distinct subclusters of MACs by DNA content (Fig. 1*D*) nor evidence for differential amplification. DNA unscrambling, a process that reorders and inverts DNA segments between IESs during development, was not directly addressed here, but has only been found in conjunction with IES elimination (13, 62), and is thus likely also absent. Likewise, to assess chromosome breakage, *Loxodes* telomeres and telomerase must be identified, but have eluded our detection (*SI Appendix*, *SI Results 13*).

### Secondary Loss or Retention of Ancestral State?

Although it is tempting to revive the theory that *Loxodes* represents a “primitive” state prior to the origin of genome editing (35, 63), it is more parsimonious to conclude that IES excision, along with dividing, ampliploid MACs, was present in the ciliate common ancestor but secondarily lost in karyorelicts, because their sister group, the heterotrichs, performs extensive genome editing with elements homologous to other ciliates (17, 20). The presence of “relict” Dcl genes in *Loxodes*, homologous to those involved in genome editing in other ciliates, also support secondary loss, whereas the apparent absence of a domesticated excisase is less conclusive, as ciliate excisases come from at least two different families (20, 24, 26, 64), and so were independently or repeatedly domesticated.

### Implications for Mobile Element Proliferation and Management.

Losing IES excision should result in MAC genomes with more mobile elements and repeats, as seen in *Loxodes*. However, it is misleading to say that genome editing “defends” against mobile elements, as often suggested (65, 66). Editing arguably helps mobile elements persist in the germline by shielding them from selection, and is maintained by evolutionary addiction rather than positive selection (16, 17, 27, 33). This interpretation can be formalized in the framework of constructive neutral evolution (CNE) (67, 68).

How does *Loxodes* ameliorate the deleterious effects of mobile elements without editing? Natural selection would eliminate the most deleterious elements, exposed in the transcriptionally active MAC. The remainder must be largely inactive or benign, such as the abundant but mostly fragmentary retrotransposon-related repeats (retrotransposons are prone to being truncated by incomplete reverse transcription) (69). Retrotransposons outnumber DNA transposons in many eukaryotes, e.g., 43% vs. 4% of the human genome (70), and are numerous in all ciliate MIC genomes examined (Fig. 4*A*) (13, 14, 16, 17, 62), but it is surprising that we have not detected compelling DNA transposon homologs in *Loxodes*. This may reflect the genomic history of this particular strain, or higher deleteriousness of DNA transposons.

*Loxodes* may also have revived or maintained ancestral eukaryotic mechanisms to suppress mobile element expression in the MAC, such as heterochromatinization, that in other ciliates have been co-opted for editing. Mature *Loxodes* MACs have the heterochromatin-associated histone mark H3K9me3 (Fig. 5*C*), which in eukaryotes generally is associated with repetitive sequences and retrotransposons suppression (71). Other ciliates exhibit H3K9me3 only in developing MACs but not mature MACs or MICs, and instead co-opt heterochromatin marks H3K9me3 and H3K27me3 to guide the editing machinery in developing MACs (3, 57), although low H3K27me3 levels have been reported in *Paramecium* mature MACs (72). As for other eukaryotic silencing mechanisms, we did not detect 5mC methylation in *Loxodes*, while the role of H3K27me3 remains unresolved as we lacked a suitable antibody.

Independently of editing, nuclear dualism itself has evolutionary consequences. Condensed MIC chromatin may hinder mobile element invasion, whereas any successful invasion of the disposable somatic MAC would not be transmitted to progeny, neither sexual nor asexual in the case of *Loxodes*. An inactive MIC also limits transcription-associated mutation, contributing to germline DNA integrity. MIC-to-MAC development in all ciliates entails considerable chromatin reorganization and DNA modifications, but the degree to which these are epigenetically inherited is unclear. *Loxodes* hence presents an opportunity to study these phenomena independently of IES excision and meiotic sex.

### Possible Scenarios for Loss of IES Excision.

The apparent loss of IES excision in *Loxodes* actually presents a challenge to the CNE model, which posits that complexity can evolve neutrally through irreversible, ratchet-like processes. By this logic, ciliates with many intragenic IESs like *Paramecium* or *Blepharisma* cannot afford to lose genome editing as the resulting erroneous retention of IESs in essential genes is likely lethal. Conversely, IESs cannot be exposed to selection in the somatic genome if they are removed by editing. How then could the *Loxodes* ancestor have lost both editing and the IESs themselves? We see three possible solutions: i) its IESs were mostly intergenic and nonlethal if retained; ii) high rates of gene duplication such that some paralogs remained undisrupted by IESs; or iii) a mature MAC without IESs was developmentally “reset” to a MIC, wiping the germline clean of IESs in one go.

Asexual MAC division was likely lost before extensive editing in karyorelicts, as the increased cost of additional MIC-to-MAC development during asexual division could cause strong selective pressure to streamline or lose genome editing. In other ciliates, MIC-to-MAC development is coupled to sex and is costly compared to asexual division because of editing, e.g., *Paramecium* requires ~22 h for sexual vs. 6 h for asexual division (73, 74). Asexual MIC-to-MAC development without prior meiosis/karyogamy has been observed in *Blepharisma*, where “somato-MICs” develop directly into MACs under some conditions (75), although genome editing is presumably still involved since its MIC genome possesses ~40,000 IESs. Karyorelict MIC-to-MAC development may stem from this “backup” somato-MIC pathway, rather than the sex-associated pathway.

The irreversible gain of genome editing is also a question in rhabditid nematodes, where it is unclear whether editing has been independently acquired multiple times, or gained once and then lost several times (76). Study of editing in these groups would add nuance to theoretical frameworks like CNE, as these have largely focused on the gain of complex traits, but not their loss.

## Materials and Methods

General reagents were analytical grade from Sigma-Aldrich/Merck unless otherwise noted. Full parameters of computational analyses are available from code repositories linked below. R.T.—room temperature.

### Isolation and Cultivation of *Loxodes* Strains.

Strains *L. magnus* Lm5 and *L. striatus* Lb1 were isolated from single cells and grown in soil extract medium as previously described (77). Both have been deposited at the Culture Collection of Algae and Protozoa (Oban, Scotland).

### Nuclei Purification by Fluorescence-Activated Nuclear Sorting.

Detailed protocol: (78). Briefly: 500 mL batches of dense, saturated cultures (*L. magnus* ~500 cells/mL, *L. striatus* ~1,000 cells/mL) were starved for at least the average doubling time of ~1 wk (77) to minimize actively dividing or developing nuclei. Cells were filtered through prewashed quartz sand, centrifuged (120 g; 2 min; R.T.) in pear-shaped glass flasks, resuspended in autoclaved Volvic water, concentrated by centrifugation to ~3 mL, then resuspended in 7.5 mL ice-cold lysis buffer (sucrose 0.25 M, MgCl_2_ 10 mM, Tris-HCl pH 6.8 10 mM, Nonidet P-40 0.2% w/v) (79) in 15 mL polypropylene tubes. The mixture (on ice) was pulled up and expelled completely five times with a 20 mL plastic syringe through a 0.60 mm × 60 mm needle to lyse cells, stained with DAPI (final conc. 1 µg/mL), transferred to 2 mL tubes, and centrifuged (2,000 g; 3 min; 4 °C). The nucleus pellet was resuspended in 2 mL ice-cold Galbraith’s buffer (MgCl_2_ 45 mM, sodium citrate 30 mM, MOPS pH 7 20 mM, Triton X-100 0.1% v/v) (80) by pipetting up and down, and kept on ice.

Suspensions were filtered through 35 µm nylon mesh “cell strainers” (Fisher Scientific 352235) and then sorted on a BD FACSMelody Cell Sorter, controlled with BD FACSChorus v1.1.18.0, with 100 µm nozzle size, 23 PSI pressure, 34.0 kHz drop frequency, and “purity” sort mode. DAPI fluorescence was measured with 405 nm laser excitation and 448/45 filter. PMT voltages were set to initial values: FSC, 300 V; DAPI, 370 V; SSC, 490 V. Populations were gated with combinations of SSC, FSC, and DAPI fluorescence (Fig. 1*D*), but exact settings were adjusted manually to account for batch variation.

Sorted nuclei were collected in 1.5 mL microcentrifuge tubes prefilled with 100 µL Galbraith’s buffer (5 °C). 10 µL samples per batch were viewed under epifluorescence microscopy (DAPI signal) to verify purity, by scoring ≥100 nuclei per sample as MIC (no nucleolus) or MAC (with nucleolus). Only samples with >99% visually verified target purity were used for downstream experiments. Collected nuclei were centrifuged (8,000 g; 3 min; 4 °C), and supernatant was removed by pipetting; pellets were snap-frozen in liquid nitrogen and stored at −80 °C until genome sequencing (*SI Appendix*, *SI Materials and Methods*) or western blotting (see below).

### RNA Library Preparation and Sequencing.

*L. magnus* cells grown in soil extract medium (77) were resuspended in fresh medium to 250 cells/mL, split into six flasks of 150 mL each, and kept at R.T. without feeding. Cell densities were monitored daily (*SI Appendix*, Table S3); three flasks were harvested for RNA extraction after 3 d (“starved” cells). The remainder were each fed 450 µL concentrated *Chlamydomonas* (77) on days 3 and 4. By day 5, dividing *Loxodes* cells were observed and cell densities began to recover, so flasks were harvested, representing “fed” cells.

To harvest, cells were filtered through cotton gauze, centrifuged in pear-shaped flasks (80 g; 1 min; R.T.), resuspended in 10 mL SMB medium in 15 mL polypropylene tubes, and centrifuged (90 g; 1 min; R.T.). Concentrated cells (~500 µL) were transferred dropwise to 3 mL ice-cold TRI reagent (Sigma-Aldrich T9424) while vortexing, and stored at −80 °C until use. For RNA extraction, thawed samples were split into 3 × 1 mL aliquots. Each aliquot was shaken with 200 µL chloroform, kept at R.T. for 2 min, then centrifuged (1,200 g; 15 min; 4 °C). Aqueous phase was transferred to new tubes, mixed with equal volume 100% ethanol, inverted 20×, then purified with Zymo RNA Clean and Concentrator 5 kit (Zymo, R1013) with in-column DNase digestion.

### Nuclei Purification for Nucleosomal DNA Sequencing.

For nucleosomal DNA sequencing, *L. magnus* cells were harvested and washed once as above, centrifuged (200 g; 1 min; R.T.), resuspended with ice-cold Galbraith’s buffer amended with bovine serum albumin (BSA, 0.05% w/v) and complete protease inhibitor (1×, Roche 11697498001), lysed by repeated pipetting, stained with DAPI (1 µg/mL) for 5 min on ice, centrifuged (500 g; 2 min; 4 °C), and resuspended again in Galbraith’s + BSA + protease inhibitor on ice. Nuclei were flow-sorted as above. Libraries were prepared with the EZ Nucleosomal DNA prep kit (Zymo D5220) (*SI Appendix*, *SI Materials and Methods*).

### Nucleosomal DNA Profiling and Phaseograms.

Nucleosomal DNA libraries for MAC and MIC (81) were mapped onto the MIC Falcon assembly (82, 83)with minimap2 v2.24 (parameter: -ax sr). Positional maps (“phaseograms”) were computed with mnutils commit 105d129 (84) (parameters: --feature gene --phaseogram --dump), using gene features predicted by Pogigwasc in GFF3 format. The insert size range (parameters --min_tlen and --max_tlen) was set to 96 to 136 bp for MAC and 126 to 166 bp for MIC, because nucleosomal DNA was more heavily digested in MAC than MIC. Read mappings without peak-calling or denoising were used to obtain a purely empirical picture of nucleosomal positioning. For all phaseograms, the midpoint of each mapped read pair was used as the nucleosomal DNA fragment position. For each mapped fragment, positions of other fragments in a 1 kbp window downstream were enumerated; the cumulative pileup of positions relative to each other constituted the global phaseogram. The Pogigwasc gene predictor only modeled coding sequences. Therefore, for the phaseogram relative to gene features, we assumed that 5′-UTR lengths are short and tightly distributed like other ciliates, and used coding sequence starts as a proxy for transcription start sites, using a window of 1 kbp on both sides. Workflow: (85).

### k-mer-Based Genomic Library Comparisons.

Adapter- and quality-trimmed (Phred score >28) Illumina reads were used for k-mer-based comparisons (86). k-mer content (k = 21) of genomic libraries (83, 87) were compared pairwise with each other, or with the reference MAC genome, using the “kat comp” command in kat v2.4.2 (88), which depends on jellyfish (89) and SeqAn (90).

### Genome Assembly.

PacBio reads were demultiplexed and processed to circular consensus sequence (CCS) reads with PacBio SMRT Link v9. Analysis of an initial assembly with Flye v2.8.1 (91) (option: --pacbio-hifi) showed that the genome was probably diploid; therefore, CCS reads were assembled again with the diploid-aware assembler Falcon (Bioconda package pb-falcon 2.2.4 installed with package pb-assembly v0.0.8) (92) using a relatively low identity threshold of 0.96 for collapsing heterozygosity (option: overlap_filtering_setting=--min-idt 96) and option: ovlp_daligner_option = -e.96. Other parameters followed the template configuration for CCS reads (https://github.com/PacificBiosciences/pb-assembly/blob/master/cfgs/fc_run_HiFi.cfg). The average coverage (~20 to 30×) was below the recommended ~30× per haplotype for phased assembly, so we did not proceed to Falcon-Unzip. Falcon primary contigs were polished with Racon v1.4.20 (93) using read mappings from pbmm2 v1.4.0 filtered with samtools view using options -F 1796 -q 20 (exclude unmapped reads, nonprimary alignments, reads that fail platform/quality checks, and PCR or optical duplicates; minimum quality Phred 20). Workflow: (94).

### Annotation of Repeats in Genome Assembly.

Low-complexity tandem repeats were annotated with TRF v4.09.1 (95), using the recommended algorithm settings: 2 5 7 80 10 50 2000 -d -h -ngs. The output was filtered and converted to GFF format with trf_utils (96), retaining repeat regions ≥1 kbp long; if features overlapped, the highest-scoring feature was retained, otherwise the feature with the most repeat copies. The filtered feature table was merged and used to mask the assembly with the merge and maskfasta commands in bedtools v2.27.1 (97).

Interspersed repeat element families were predicted from the MIC genome assembly with RepeatModeler v2.0.1 (default settings, random number seed 12345) with the following dependencies: rmblast v2.10.0+ (http://www.repeatmasker.org/RMBlast.html), TRF 4.09 (95), RECON (98), RepeatScout 1.0.6 (99), RepeatMasker v4.1.1 (http://www.repeatmasker.org/RMDownload.html). Repeat families were also classified in the pipeline by RepeatClassifier v2.0.1 through comparison against RepeatMasker’s repeat protein database and the Dfam database. Predicted repeat families were annotated in both the MAC and MIC assemblies with RepeatMasker, using rmblast as the search engine.

### Transcriptome Mapping and Assembly.

RNA-seq libraries (100) were adapter- and quality-trimmed (Phred > 28, length ≥ 25 bp) with bbduk.sh from BBtools v38.22, and mapped with bbmap.sh (BBtools) to the *Chlamydomonas reinhardtii* genome (JGI Phytozome assembly v5.0, annotation v5.6) (101) (identity ≥ 0.98) to remove potential contamination from food algae. RNA-seq reads were mapped to *Loxodes* assemblies with Hisat2 v2.0.0-beta (102), modified to lower the minimum allowed intron length to 10, with options: --min-intronlen 10 --max-intronlen 50000 --seed 12345 --rna-strandness RF. Workflow: (94).

### IES Prediction.

PacBio CCS reads (83) were mapped to the MAC Falcon assembly with minimap2 v2.17 (103) with the options: --MD -ax asm20. BAM files were sorted and indexed with samtools v1.11 (104). Putative IESs were predicted from the mapping BAM file with BleTIES MILRAA v0.1.11 (42) in CCS mode with options: --min_break_coverage 3 --min_del_coverage 5 --fuzzy_ies --type ccs, parallelized with ParaFly commit 44487e0 (https://github.com/ParaFly/ParaFly). Workflow: (105).

### Variant Calling and Comparison to Putative IESs.

Variants were called with Illumina short reads (more accurate, higher coverage), whereas phasing and haplotagging were performed with PacBio long reads, as recommended in the WhatsHap documentation. Illumina MIC and MAC reads (83) were mapped to MAC reference assembly with bowtie2 v2.3.5 (106) with default parameters. Variants were first called from mapped Illumina reads with FreeBayes v1.3.2-dirty (107) in “naive” mode to verify ploidy (options: -g 400 --haplotype-length 0 --min-alternate-count 1 --min-alternate-fraction 0 --pooled-continuous), filtered with vcffilter from vcflib v1.0.0_rc2 (108) to retain variant calls with Phred quality score >20. Variants were then called again in diploid mode (default options except: -g 400). Mapped PacBio HiFi reads were phased and haplotagged with WhatsHap v1.4 (109), using only SNPs (default). VCF files were processed (e.g., merging, indexing) with bcftools v1.11 (104). Reads with/without “IES” indels predicted by BleTIES were compared with their respective haplotags by parsing the haplotagged reads. The script used the pybedtools (97, 110) and pysam (111) libraries. Workflow: (105).

### Gene Prediction with Pogigwasc.

Introns were empirically annotated from RNA-seq mappings as they were too short to model effectively, as previously observed with *Blepharisma* (20). Introns were identified from Hisat2 mappings of RNA-seq reads vs. the MAC and MIC Falcon assemblies with Intronarrator (commit b6abd3b, https://github.com/Swart-lab/Intronarrator, options: MIN_INTRON_RATIO=0.2, MIN_INTRONS=10, MAX_INTRON_LEN=40), then removed to produce an artificial “intronless” assembly; noncoding RNAs identified with Infernal v1.1.4 (112) were hard-masked. Scaffolds were split to contigs on gaps and hard-masked sequences; contigs <1 kbp were removed. Protein coding sequences were predicted from the resulting “intronless” contigs with Pogigwasc v0.1 (49) (option: --no-introns) using parameters trained on *L. magnus*, which are bundled with the software. Annotations were translated back to original genomic coordinates with pogigwasc-utils (113) (commit 7844e1). Gene predictions overlapping with low complexity regions predicted by TRF (see “*Annotation of Repeats in Genome Assembly*”) were identified with bedtools intersect (options: -v -f 1.0). Workflows: (114, 115).

### Functional Genome Annotation and Screening for Genome Editing Toolkit.

The *L. magnus* predicted MIC and MAC proteomes from Pogigwasc, MAC proteomes from 13 ciliate species, and translated ORFs >30 a.a. predicted by getorf (EMBOSS v6.6.0.0) from 4 species’ MIC genomes (*SI Appendix*, Table S4), were annotated with InterProScan v5.57-90.0 (116). Protein domains, signatures, and motifs relevant to the following were shortlisted by InterPro database keyword searches (117): DNA transposons and retrotransposons, Dicer and Dicer-like proteins, and histones (118). For retrotransposons, Pfam domains not relevant to mobile elements (e.g., telomerase reverse transcriptase) were excluded: PF00026, PF12009, PF11474. To account for genes possibly missed by Pogigwasc, ciliate domesticated excisases were aligned against the *L. magnus* genome assembly with TBLASTN (Blast+ v2.12.0) (119): PiggyMac homologs from *P. tetraurelia* (Pgm, ParameciumDB PTET.51.1.P0490162), *Tetrahymena tetraurelia* (Tpb2p, Ciliate.org TTHERM_01107220), *B. stoltei* (BPgm, ciliates.org BSTOLATCC_MAC17466), TBE element excisase from *Oxytricha trifallax* (Genbank AAB42034.1).

### Antibody Detection of Histones, Histone Marks, and 6mA Base Modification.

Commercially available primary antibodies were used against: acetyl histone H3 lysine 9 (H3K9ac), trimethyl histone H3 lysine 9 (H3K9me3), trimethyl histone H3 lysine 4 (H3K4me3), total histone H3, total histone H4, and 6mA base modification (*SI Appendix*, Table S5). Antibodies were applied to flow-sorted nuclei for western blotting, and to whole cells for immunofluorescence (protocol adapted from ref. 77, and from ref. 58 for 6mA) (*SI Appendix*, *SI Materials and Methods*).

Western blotting with two additional antibodies was not successful: anti-trimethyl histone H3 lysine 27 (H3K27me3, Merck 07-449) (its 6 a.a. immunogen sequence was not found in *Loxodes* histone H3), and anti-histone H4 (Santa Cruz sc-25260) raised against human histone H4.

### 6mA Base Modification Analysis from PacBio SMRT-Seq Reads.

PacBio SMRT-Seq subreads for flow-sorted MAC and MIC DNA were indexed with pbindex (PacBio SMRT Link v12.0.0) and Falcon assemblies with samtools faidx. Subreads were aligned to respective assemblies with pbmm2 (SMRT Link v12.0.0), a modified minimap2 version (103), using parameters “align --preset SUBREAD.” 6mA modifications were identified with “ipdSummary” in kineticsTool (SMRT Link v12.0.0), with parameters “--identify m6A,m4C,m5C_TET --methylFraction,” excluding mitochondrial contigs (1 MIC, 4 MAC), at ≥25× subread coverage and identification quality ≥30 (*SI Appendix*, *SI Materials and Methods*). Genes ≥ 1,000 bp were selected to assess 6mA levels across gene bodies. The same methods and thresholds were applied to call 6mA in MAC read data of *B. stoltei* (20).

We did not detect cytosine methylation, which has been reported in some ciliates but apparently absent in others. No canonical cytosine DNA methyltransferases have been identified yet in ciliates (5, 120, 121); we also did not detect them in *Blepharisma*.

## Supplementary Material

Appendix 01 (PDF)

## Data Availability

Software is archived on Zenodo (49, 84–86, 94, 96, 105, 113–115, 122). Sequencing data are deposited in the European Nucleotide Archive (ENA) (81, 83, 87, 100). Flow cytometry data (123–127), Western blots (128), immunofluorescence imaging (129), *L. magnus* genome annotations (82), and variant calling (130) are deposited in EDMOND (Max Planck Digital Library).
