# Supplementary material for: Nuclear dualism without extensive DNA elimination in the ciliate Loxodes magnus
Source: Proc Natl Acad Sci U S A. 2024 Sep 19;121(39):e2400503121. doi: 10.1073/pnas.2400503121 (PMC11441545; doi:10.1073/pnas.2400503121)
Supplement: Supplementary file 1 — Appendix 01 (PDF) [file pnas.2400503121.sapp.pdf]

PNAS

Supporting Information for

Nuclear dualism without extensive DNA elimination in the ciliate *Loxodes magnus*.

Brandon K. B. Seah, Aditi Singh, David E. Vetter, Christiane Emmerich, Moritz Peters, Volker Soltys, Bruno Huettel, and Estienne C. Swart

Brandon K. B. Seah
Email: kb.seah@gmail.com

Estienne C. Swart
Email: estienne.swart@tuebingen.mpg.de

This PDF file includes:

SI Materials and Methods
SI Results
SI Discussion
Figures S1 to S16
Tables S1 to S5
SI References

SI Materials and Methods

Genomic DNA library preparation and sequencing. DNA was isolated from sorted nuclei with the CleanNA Clean Blood and Tissue kit (CBT-D0096), resuspended in 10 mM Tris-HCl pH 8.5, and quantified with the Qubit DNA High Sensitivity kit. Short-read libraries (two replicate batches per nucleus type per species) were prepared with the NEBNext Ultra II FS DNA Library Prep kit (NEB E7805), and sequenced 150 bp paired-end on an Illumina HiSeq3000. Long-read libraries (one per nucleus type for L. magnus only) were prepared with the SMRTbell Express Template Prep Kit 2.0 using the Sequel II Binding Kit 2.0 with Sequel polymerase 2.0, size-selected to 10 kbp, and sequenced with the HiFi protocol on a PacBio Sequel II.

Control against inadvertent cross-mixing of sorted nuclei or reads. The *Loxodes magnus* MAC library had slightly more unique k-mers (2.0%) than the MIC library (1.3%), which may represent low-abundance contaminants, e.g. bacteria or feed algae. Sorted samples were largely free of bacterial contamination: in short read libraries prepared from sorted MIC and MAC libraries, at most 1.3% of reads mapping to SSU rRNA sequences were classified as bacterial or archaeal (Figure S2). There was no single predominant non-*Loxodes* taxon, so the expected coverage of any single contaminant was negligible.

To account for inadvertent cross-mixing during pooling of sorted nuclei or reads, two distinct sorted samples of each type of nucleus (and corresponding read libraries) were prepared per species. k-mer content was compared for each pairwise combination of libraries in each species. Pairwise comparison of individual replicates showed the same pattern as for the pooled libraries described above, both for *L. magnus* (Figure S3B) and *L. striatus* (Figure S4B), indicating that cross-mixing of nuclei types during pooling was unlikely.

Genome assembly from short reads. Illumina reads were trimmed to remove sequencing adapters, phage Phi X-174 sequence, and low-quality bases (Phred score < 28) with bbduk.sh from the BBTools software package v38.22 (http://sourceforge.net/projects/bbmap/), with options: ktrim=r qtrim=rl trimq=28 minlength=25. Trimmed reads were then assembled with SPAdes v3.13.0 (1) with options: --memory 380 -k 21,33,55,77,99,127.

Genome assembly quality control. Illumina genomic DNA read libraries were screened for SSU rRNA genes with phyloFlash v3.1 (2), using the SILVA 132 SSURef NR99 database (3), to screen for potential contaminants. “Blob plots” of average contig coverage vs. contig GC% were plotted to visualize contig clusters that may represent contaminants. Most *Loxodes* contigs had ~25% GC, like most ciliate nuclear genomes; a contig cluster with ~45% GC was found to largely comprise low-complexity tandem repeats (Figure S6).

Genome completeness was estimated from proteins predicted in the *L. magnus* MAC genome by Pogigwasc (see main text Methods, “Gene prediction with Pogigwasc”), alongside 13 published ciliates and the alveolate *Perkinsus* marinus as outgroup (Table S4), with the Alveolata marker set in Busco v5.0.0 (4, 5) using parameters: -m protein -l alveolata_odb10.

Simulation of k-mer comparisons for MIC and MAC of *Paramecium tetraurelia*. *Paramecium tetraurelia* strain 51 MAC and MAC+IES genomes (v1.0) were obtained from ParameciumDB (https://paramecium.i2bc.paris-saclay.fr/files/Paramecium/tetraurelia/51/).(6) 10 million 150 bp paired-end reads were simulated from each library (random seed 0) with randomreads.sh from the BBTools software suite v38.18 (https://sourceforge.net/projects/bbmap/). A second MAC library was simulated with random seed 54321. Mixed libraries were simulated by subsampling the simulated libraries with reformat.sh from BBTools, using sample seed 12345 and sample rates 0.1, 0.3, 0.5, 0.7, and 0.9, then concatenating the respective subsamples in the required proportions. k-mer frequencies were compared with kat comp from Kat v2.4.2 (7) using k-mer length 19.

sRNA library preparation and sequencing. Input RNA was quality assessed by capillary electrophoresis (small RNA chip, Agilent Bioanalyser); an Illumina-compatible NGS library was prepared with a NEXTflex Small RNA-Seq Kit v3 (Bioo Scientific) and sequenced in 2 x 150 bp paired-end read mode on an Illumina HiSeq3000 instrument at the Max Planck Genome Centre Cologne.

sRNA-seq analysis. sRNA reads were trimmed with Trim Galore v0.6.7 (10.5281/zenodo.5127898) with parameters "--fastqc --small_rna --length 10 --paired", merged with bbmerge (8) (parameters "mininsert=10 mininsert0=10 minoverlap=10), then mapped to the *Chlamydomonas reinhardtii* genome with bbmap (parameter: "k=10") from BBTools v38.22 (9). Unmapped reads were mapped to the *Loxodes magnus* MAC and MIC genomes with bbmap and the same parameters. To generate length/5' base composition histograms, replicates within the "fed" and "starved" conditions were combined since the individual replicate histograms were similar. Fed cell sRNAs appeared to be subject to less degradation of larger RNAs, as judged by the sRNA length histograms, and thus we focused on their properties.

Workflow: https://github.com/Swart-lab/loxodes-srna-workflow

Transcriptome assembly. For initial parametrization of the gene prediction model, a de novo transcriptome was assembled by mapping-guided assembly with Trinity v2.11.0 (10), using the preliminary SPAdes MAC genome assembly as a reference, with options: --SS_lib_type RF --genome_guided_max_intron 200.

Workflow: https://github.com/Swart-lab/loxodes-assembly-workflow

RNA-seq count estimation. GFF3 output from Pogigwasc was converted to GTF by GffRead (11), then all "CDS" annotations were substituted with "exon" annotations. TPMCalculator (12) version 0.0.4 with optional switches "-c 14 -p -a" was then used to estimate TPM values across all the genes in the *Loxodes* MAC genome, using a BAM file of all the pooled RNA-seq from both starved and fed cells mapped to this genome.

Nucleosomal DNA library preparation and sequencing. Sorted nuclei were centrifuged (1000 g; 2 min; 4 ºC), and supernatant removed by pipetting. Each nuclei pellet was washed with 100 µL ice-cold Atlantis digestion buffer, centrifuged (200 g; 1 min; 4 ºC), resuspended in cold 50 µL Atlantis digestion buffer by 10× repeated pipetting, then incubated with 10 units Atlantis dsDNase (42 ºC; 40 min). Stop solution was added, then DNA was purified on spin columns and eluted in 30 µL buffer. Mono- and di-nucleosomal DNA fragments (~150 to 300 bp) were size-selected with SPRIselect magnetic beads (Beckman-Coulter B23317) using "right size selection" and 0.7× beads:sample volume ratio. Fragments were sized with a Bioanalyzer 2100 DNA high sensitivity assay (Agilent 5067-4626). Libraries (two replicates per nucleus type) were prepared with the NEBNext Ultra II DNA library prep for Illumina kit (NEB E7645S) and sequenced on an Illumina NextSeq2000.

rDNA searches and HiFi CCS read mapping to representative rDNA locus. rDNA searches were performed in the *Loxodes magnus* MAC and MIC genome assemblies using Infernal (13) with default parameters using the relevant models from RFAM (14) for the 5S, SSU, 5.8S and LSU rRNAs.

PacBio SMRT-seq HiFi CCS reads for the flow sorted MAC and MIC DNA were aligned to a complete representative rDNA sequence extracted from the *Loxodes magnus* MAC genome (as defined by a search of RFAM using Infernal) using pbmm2 (15), from the SMRT Link v12.0.0 software bundle from Pacific Biosciences (command ""pbmm2 align --preset CCS"; after indexing the reads with "pbindex").

Western blotting of flow-sorted nuclei. Nuclear pellets from flow sorting were resuspended with 1× protein loading buffer (PLB, 100 mM Tris-HCl pH 6.8, 4% (w/v) sodium dodecyl sulfate, 20% (w/v) glycerol, 0.2 M dithiothreitol, 0.05% (w/v) bromophenol blue) diluted with PBS (1000 nuclei per 1 µL final volume), and heated (95°C, 10 min). For each lane, 10 µL of sample in PLB was loaded onto a 12% SDS-PAGE gel and separated (200 V; 45 min) on a Bio-Rad Mini-Protean Tetra Cell electrophoresis system. Silver staining was performed with the Pierce Silver Stain Kit (24612, Thermo Fisher Scientific). For Western blots, proteins were transferred (80 V; 2 h; 4°C) onto a 0.2 µm nitrocellulose membrane (Bio-Rad 1620112). Membranes were air-dried, blocked with 5% (w/v) Bovine Serum Albumin (BSA) (Sigma A9647) with 0.2% (v/v) Tween-20 (Sigma P2287) in PBS (overnight; 4°C), incubated with primary antibodies diluted in 5% BSA / 0.2% Tween-20 / PBS (overnight, R.T.), washed in 0.2% Tween-20 / PBS (3 × 10 min), incubated in the secondary antibody horseradish peroxidase (HRP)-conjugated goat anti-rabbit IgG (Merck 12-348) (1 h; R.T., washed with 0.2% Tween-20 / PBS (3 washes × 10 min), then washed in PBS (5 min). 200 µL of chemiluminescence substrate (Immobilon Crescendo Western HRP, Millipore, WBLUR0100) was added to each membrane, which was then imaged on a AI600 imager (GE Healthcare).

For Coomassie staining, 10 µL of resuspended protein samples in PLB were loaded on a 12% SDS-PAGE gel; samples were run in 1× Laemmli Buffer (Tris-base, Glycine, SDS) at 180 V until the loading dye ran out of the gel. The gel was stained with Coomassie blue (PhastGel blue R, Sigma, 6104-59-2) (overnight on orbital shaker; R.T., removed from staining solution, washed with autoclaved double distilled water (2 x 5 min), destained (25% v/v isopropanol, 10% v/v acetic acid in deionized water) until protein bands were clearly visible, then imaged with an AI600 imager.

Immunofluorescence for histones, histone marks, and 6mA base modification. 100 mL of dense culture was centrifuged in pear-shaped flasks (80-120 g; 1 min; RT), resuspended in SMB medium to wash, centrifuged again, resuspended in 500 µL of SMB medium, and fixed at R.T. with an equal volume of ZFAE fixative.(16) Subsequent transfers were performed by centrifugation (1000 g; 1 min) followed by removal of supernatant and resuspension of pellet at R.T. Fixed cells were permeabilized 5 min in 1.5 mL 1% (w/v) Triton-X / PHEM, post-fixed 10 min in 1 mL 2% (w/v) formaldehyde / PHEM, then washed twice for 5-15 min in 1 mL 3% (w/v) BSA / TBSTEM. Antibodies were diluted to working concentrations (Table S5) in 3% BSA / TBSTEM. The secondary antibody was Alexa Fluor 568-conjugated goat anti-rabbit IgG (Life Technologies, A11011).

For histones and histone marks, fixed cells in BSA were incubated 10-60 min in primary antibody working solution, washed 5-10 min in 3% BSA / TBSTEM, then incubated 10-30 min in the secondary. Cells were counterstained ≥5 min with DAPI (1 µg/mL in 3% BSA / TBSTEM), mounted under ProLong Gold (Thermo Fisher), and cured (overnight; R.T.), then imaged by epifluorescence on a Zeiss AxioImager Z1 (Plan-Apochromat 63×/1.40 oil objective, Axiocam 702 camera, filter cubes Zeiss 49 for DAPI and AHF F46-008 for Alexa Fluor 568).

For 6mA, *Loxodes magnus* was harvested, fixed, permeabilized, post-fixed, washed, and resuspended in 3% BSA/TBSTEM as described above. The following was adapted from (17): Fixed cells were treated with RNase A (50 µg/mL; 2 h; 37 °C), resuspended in 2 M HCl (20 min; R.T.), washed with 1 M Tris-HCl pH 8, resuspended in 3% BSA/TBSTEM, incubated with primary antibody (Table S5; overnight; 4 °C), washed with BSA/TBSTEM, then incubated with secondary antibody (30 min; R.T.). Cells were counterstained with DAPI, mounted, and imaged as described above.

Choice of 6mA detection threshold. At the subread coverage threshold of 25, Pacific Bioscience's base modification detection software reported 1.13% of adenosines in the *L. magnus* MAC genome assembly as 6mA (5,870,562 potential 6mA positions out of 520,983,087 adenosines), compared to 0.0157% of adenosines in the MIC genome assembly as 6mA (98,290 potential 6mA positions out of 626,673,708 adenosines). However, the modification identification quality values ($-10\times\log_{10}$(probability of incorrect detection)) reported for the MIC genome in general were much lower than for the MAC genome (median 8 vs. median 64). Thus, the percentage of 6mA bases called in the genomes was estimated at ≥ 25× coverage and an identification quality value ≥ 30.

Of the 32,407 adenosines in a ~76 kb *L. magnus* mitochondrial contig (CAMPDZ020002550) co-assembled from residual DNA present in the MIC fraction, just one adenosine base was classified as 6mA at this threshold. This base was not followed by the characteristic thymine base and was called with an identification quality value of 5, and is likely thus a false positive modification call.

SI Results

1. Simulation of k-mer comparisons for MIC and MAC of *Paramecium tetraurelia*. If the MIC genome contains germline-limited IESs, which in other ciliates can represent ≥10% of the total MIC sequence content, we would expect to see unique k-mers in the MIC library, with a k-mer frequency peak similar to the main genome peak of shared k-mers. Contamination of the MAC library with MIC sequences would reduce the number of unique k-mers, but the MIC-specific k-mers should still be recognizable as a distinct cluster whose frequencies differ between the MIC and MAC libraries, e.g. in a MIC vs. MAC k-mer frequency heatmap, as long as the target genomes have been enriched.

To explore the expected results of k-mer comparison when libraries have different degrees of contamination (i.e. MAC contaminated with MIC and vice versa), we simulated short-read shotgun sequencing libraries of MIC and MAC genomes from reference assemblies of *Paramecium tetraurelia* strain 51. The *P. tetraurelia* MAC+IES assembly was used in lieu of the published de novo MIC assembly because of its higher contiguity. Published real sequencing data from ciliate genomes were not suitable for such benchmarking because the expected purity is unknown, and there is often also contamination from other organisms, e.g. bacteria.

When two pure MAC libraries with high coverage are compared, the only source of unique k-mers should be sequencing errors, visible as a left-sloping curve in the k-mer frequency spectra (Figure S14). Most k-mers in the k-mer comparison heatmap should fall along the 1:1 line (Figure S15). The spectra show multiple peaks because *P. tetraurelia* has experienced several whole genome duplication events in its evolutionary history.

When a pure MAC library is compared against a mixed MAC+MIC library, the k-mers originating from the IESs of the MIC library are noticeable in the frequency curve of k-mers unique to the mixed library (Figure S14, dashed orange lines), which have frequencies above the background sequencing error k-mers. The MIC-specific unique k-mer peak is apparent even when the MAC content of the MAC+MIC library is as high as 50-70%. As the fraction of MIC in the mixed library is increased, the frequency peak of these unique k-mers also increases correspondingly. Finally, when a pure MAC library is compared with a pure MIC library, the frequency peaks of both the unique and shared k-mers should align with the overall average k-mer coverage (Figure S14).

In k-mer frequency heatmaps, the MIC-specific k-mers are visible as clusters off the 1:1 axis (Figure S15). Although the difference between MIC and MAC libraries is characterized as MIC-specific IESs, there is also a cluster of MAC-specific k-mers, which corresponds to the sequences at IES junctions after excision.

If both libraries are impure, MIC- and MAC-specific k-mers should nonetheless be detectable as unique k-mers in the k-mer frequency spectra (Figure S16A), and as off-axis clusters in the k-mer comparison heatmaps (Figure S16B), even when only 70% of each library is composed of the target genome.

Despite IESs only comprising 3.6 Mbp total sequence, compared with the total MAC assembly size of 72.1 Mbp (MAC+IES assembly 75.7 Mbp), differences in the two libraries were readily observable in the k-mer comparisons even with moderate cross-contamination.

In most ciliates, the challenge is to enrich sufficient MIC DNA because the overwhelming majority of genomic DNA in the cell is in MACs, due to somatic genome amplification. In *Paramecium*, for example, MIC abundance prior to sorting was estimated at 3% (18). In contrast, *Loxodes* has MACs with DNA content about 100-200% that of MICs, substantially less than other ciliates. In *Loxodes magnus* and *L. striatus*, there are similar numbers of MIC and MAC per cell, so the initial (unsorted) germline genome coverage is already between 33-50%. Our checks of sorted nuclei by microscopy found >99% purity based on the presence/absence of nucleoli in MACs vs. MICs respectively. Presence of a nucleolus is an unambiguous marker of a mature, functional MAC, but for the MIC fraction, there remains the possibility some nuclei that appear to be MICs by morphology or chromatin characteristics may in fact be developing MACs, even though we took measures to minimize the number of dividing cells in the cultures that were harvested for nuclei purification (cultures at saturation density, starved for ~1 week before harvesting). Even in this scenario, each cell must have at least one true germline nucleus by definition. Each *L. magnus* cell has 10-20 apparent MICs, and each *L. striatus* has two, so the minimum true germline

genome coverage in the sorted MIC fractions must be 5-10% and 50% respectively, even if some apparent MICs are actually developing MACs.

2. Hundreds of rRNA genes are encoded in the *L. magnus* assembly, often as extended tandem arrays, and are not amplified during MAC genome development. Most *Loxodes magnus* rDNAs are in tandem head-to-tail arrays distributed across both MAC and MIC genome assemblies (annotations available at: https://doi.org/10.17617/3.9QTROS). This organization is a common one in other eukaryotes, and was presumably inherited from the eukaryotic common ancestor (19). We observed roughly four arrangements of rDNA in *Loxodes magnus*: (i) isolated genes; (ii) dense clusters of tandem rDNAs at the ends of the assembled sequences; (iii) assembled sequences that were mostly or almost entirely composed of tandem rDNA repeats; (iv) rDNA tandem repeats with regular spacers between the rDNAs that are tens of kilobases in length. We did not observe substantial differences in MIC and MAC rDNA arrangements.

The dense clusters of rDNAs at the ends of some of the assembled *L. magnus* sequences resemble subtelomeric rDNA arrays found in diverse eukaryotes including the thaustochytrid *Aurantiochytrium* (20), *Encephalitozoon intestinalis* (21), the yeast *Yarrowia lipolytica* (22) and in *Giardia lamblia (23)*. The largest and densest clusters of rDNAs in *Loxodes* were either located at the ends of the assembled genomic sequences or comprised most of them. So, it is possible that *Loxodes* may have substantial subtelomeric rDNA clusters that have not yet been completely assembled.

For the rRNA 5S, 5.8S, small subunit (SSU) and large subunit (LSU), selecting only the lowest E-value matches (E-value <= 1e-22, 1e-25, 0 and 0, respectively) from Infernal, we obtained the following numbers MAC: 5S – 343, 5.8S – 405, SSU – 457, LSU – 588; MIC: 5S – 358, 5.8S – 495, SSU – 553, LSU – 676. To put these numbers in perspective, they are the same order of magnitude as those in the human genome (24). 5S rRNA genes are encoded at different locations to the other rRNA genes. The fact that the number of 5S genes (transcribed by RNA Polymerase III) is of the same order of magnitude as the other rRNA genes (transcribed by RNA Polymerase I) in both genomes is consistent with rDNA being chromosomal rather than extrachromosomal in MACs as in model ciliates like *Tetrahymena*, *Paramecium* and *Oxytricha*.

To assess whether rDNA genes may be more amplified in the *L. magnus* MAC genome as it is in ciliates like *Oxytricha trifallax* (56× mean nanochromosome copy number) (25) and *Tetrahymena pyriformis* (200× per MAC genome copy) (26), we mapped all the HiFi CCS reads underlying our genome assemblies to a single, complete, representative rDNA locus (4.9 kbp). rDNA coverage was similar in the MAC (mean 5119, s.d. 385) and MIC (5817, s.d. 347). The total quantity of input CCS reads used in assembly and mapping was 6.6 Gbp and 6.1 Gbp respectively for the MAC and MIC genome libraries. Thus, contrary to the past proposal for *Loxodes* (27), there is no evidence of amplification of MAC rDNA.

Given our observations of similar organization and rDNA copy number between MIC and MAC genome assemblies, we think it likely that the sequences that are almost entirely rDNAs in either assembly are not extrachromosomal DNA, but rather simply incomplete sequences where the assembler prematurely stopped.

3. Putative "IESs" are monoallelic indel polymorphisms. Differences between MIC and MAC libraries may not be obvious from high-level k-mer comparison if sorting of nuclei was imperfect, or if IESs constituted only a small percentage of the total genome. Additionally, if the MAC library was contaminated by some MIC sequence, the resulting MAC assembly may include some IESs. However, we reasoned that IESs should still be identifiable from read mappings, so long as there was significant differential enrichment in the samples. They should appear as inserts relative to the MAC reference genome, with higher coverage of the insert sequence in the MIC-enriched than MAC-enriched library. Given that the ratio of MIC:MAC in *Loxodes magnus* cells is between 1:1 to 1:2 to begin with, flow sorting should yield significantly >50% of the respective targets in the sorted nuclei libraries.

Based on previous analyses of MIC-enrichment libraries of other ciliate species, we expected the following: (a) More IESs should be predicted from the MIC-enriched than the MAC-enriched library; (b) IES retention scores, i.e. IES coverage relative to flanking sequences, should be >0.5 in the MIC library and <0.5 in the MAC library; (c) IESs predicted from the MIC library should be predominantly insertions relative to the MAC reference.

“IESs” from *L. magnus* were probably monoallelic indel polymorphisms in a diploid genome, because an average retention score of ~0.5 in both MIC and MAC libraries means that reads with vs. without the “IES” have ~1:1 coverage ratio. (Biallelic polymorphisms cannot be detected as the data come from a single clonal strain.) We use the term “indel” here to refer to any insertion/deletion, regardless of length or mechanism of origin, including mobile element insertions. We first verified that the *L. magnus* genome is diploid heterozygous: single nucleotide polymorphisms (SNPs) called on the MAC assembly were represented by two variants per site at ~1:1 average coverage (Figure S5A, S5B). “IES” indels in HiFi long reads were often correlated with SNP variants in the same reads, regardless of whether they were from the MIC or MAC libraries (Figure 2C). About 10% of “IES” indels were covered by at least 2 haplotagged reads with and without the indel, and so could be used to test this systematically. Of these, the majority (60-70%) were consistently associated with SNP-based read haplotypes (Figure S5C, S5D), i.e. are likely to represent monoallelic indel variants. Nonetheless, more indel polymorphisms were bound by terminal direct repeats (TDR) than expected by chance, especially TDRs that contain TA-submotifs (Figure 2E), and hence could have originated from mobile elements.

For comparison, a similar number of sequence variants were called from MAC and MIC Illumina libraries mapped to the MAC Falcon assembly with a conventional variant caller, Freebayes, in diploid mode. MAC: 437,822 SNPs, 39,048 multi-nucleotide polymorphisms, 42,836 indels. MIC: 437,157 SNPs, 38,966 multi-nucleotide polymorphisms, 42,657 indels. The median indel length in both libraries was 1 bp.

4. Genome assembly, gene prediction, and genome completeness. We expected the *Loxodes magnus* genome to have high heterozygosity because the strain used was recently isolated (August 2018) and to our knowledge has not undergone selfing. High repeat content was also expected, from the k-mer spectra of short read libraries. Therefore, we sequenced long-read libraries (PacBio HiFi error-corrected) for de novo assembly of *L. magnus* MIC and MAC genomes.

Long-read assemblies were >10-fold more contiguous than short-read assemblies (N50 ~200 kbp vs. ~10 kbp respectively, Table S1), despite a lower average coverage of 20-30×. Both Flye and Falcon assemblers gave similar contiguity. Because of the expected heterozygosity, we chose the assembly from the diploid-aware Falcon assembler (28), used a relatively low threshold to collapse heterozygosity, and polished primary contigs with Racon (29).

Published gene prediction software tools assume that stop codons are deterministic. We therefore modified a generalized hidden Markov model (GHMM) for eukaryotic genes (30) to accommodate ambiguous stop codons, implemented in the Java software package Pogigwasc (https://github.com/Swart-lab/pogigwasc). Technical details of the model and implementation are described in (31). Briefly, genome sequence is modeled as a sequence of the following hidden states (Figure 3A): Upon initiation, the model enters non-coding sequence (NCS) state; NCS emits 1 nt, then either loops back to NCS, or enters forward-strand Start or reverse-strand Stop states; genes can be encountered in either orientation, and are correspondingly represented by two sets of states. “Start” emits a 3 nt Kozak consensus sequence followed by a deterministic AUG start codon, then enters coding sequence (CDS) state. “CDS” emits 3 nt (one codon), then either loops back to CDS or enters “Stop” state. To avoid overfitting, codon emission probabilities follow a simplified model where the three codon positions are assumed to be independent, each drawing from the four possible nucleotide probabilities. “Stop” emits 24 nt, comprising 21 nt (seven codons) where the codon UGA is forbidden, followed by the UGA stop codon, then enters the NCS state. An intron model in the software was not used in this study.

To train and test the model, genes were manually annotated on the SPAdes preliminary assembly, based on alignments with assembled poly-A-tailed transcripts, mapping of RNA-Seq reads, and BLASTX alignments to other ciliate proteins. 152 genes were used for model training and 52 for testing.

201,931 protein-coding genes were predicted in the *L. magnus* MAC reference genome (Figure 3F), excluding predictions that overlapped with low-complexity regions. Of these, 255 contained introns with conflicting orientation and were also excluded. Genes had mean length 949 bp, although the longest prediction of 89 kbp was likely spurious. The majority were single-exon (95%) or had only one intron (3.3%), and most of the empirically defined introns (15,311 of 17,841) were contained within gene predictions. Visual inspection of RNA-seq mapping revealed errors, especially misplaced start or stop sites, which could account for the short average gene length (Figure 3F). Nonetheless, coding sequence appeared to be largely annotated, as the predicted proteome had a completeness of 94% based on 160

of 171 conserved marker genes for Alveolata identified by BUSCO, of which 108 were represented by a single copy. The completeness compared favorably to other published ciliate genomes (83 - 100%, excluding the outlier *Euplotes vannus*; Figure S9).

5. Dicer and Dicer-like proteins in *Loxodes magnus*. Dicer proteins (Dcr) are canonically involved in the RNA interference pathway, whereas the related Dicer-like proteins (Dcl) are specific to ciliate genome editing. Both Dcr and Dcl proteins each have a pair of Ribonuclease_III domains (PF00636), but Dcrs have additional N-terminal domains ResIII (PF04851), Helicase_C (PF00271), and Dicer_dimer (PF03368). Like other ciliates, *L. magnus* encodes both Dcrs and Dcls that cluster with their respective counterparts in a tree of the ribonuclease_III domain, and the clusters also correlate with the presence of expected domains (Figure S10).

6. Search for development specific small RNAs in *Loxodes magnus*. Ciliate small RNAs (sRNAs) are typically 20-30 nt long (32–36), and multiple sRNA classes may be expressed, e.g. several classes of small RNAs are produced during *Paramecium* development, two of which, scnRNAs and iesRNAs, are development-specific and involved in IES excision (33). The shortest class of sRNAs in *Paramecium*, siRNAs (mostly 23 nt), are not development-specific but instead regulate gene expression by silencing (37, 38).

sRNAs from both starved or actively growing *L. magnus* cells had peaks in their length distribution at 24 and 25 nt. A strong 5' U bias was present in 24 nt sRNAs (81% U), with a weaker one, closer to parity with A in 23, 25 and 26 nt sRNAs (53-55% U; Figure S11A; genomic DNA A=T=37%). This could either be due to overlapping sRNA classes in this size range or to differential, length-dependent processing by the enzymes that produced these sRNAs.

Inspection of contigs with the most mapped 24-26 nt sRNAs revealed clusters predominantly mapping to one strand or the other (e.g. Figure S11B; 24 nt sRNAs — 301 of 304; 25 nt sRNAs — 835 of 839). Thus these sRNAs resemble antisense siRNAs from *Tetrahymena (39)* and *Paramecium*, which are products of an RNA-dependent RNA Polymerase (RdRP),(38) rather than development-specific scnRNAs and iesRNAs which should map to both DNA strands (33, 40). Thus, the sequenced sRNAs in vegetative *L. magnus* cells are likely siRNAs that regulate gene expression rather than assist DNA elimination. However, given the presence of multiple Dicer-like proteins, it is conceivable that *L. magnus* produces conjugation-specific sRNAs with roles unrelated to DNA elimination.

We inspected one cluster of sRNAs with an underlying gene in detail. We selected this cluster because it encodes a protein with convincing homologs detectable by BLASTP in GenBank's nr database of similar length proteins (~620 aa; *L. magnus* gene ID: 000045F.g151_trans; e.g. to GenBank accession: KAJ3017585.1; E-value 7e-118; search conducted on 26.07.2023). The protein this gene encodes has a protein tyrosine and serine/threonine kinase (PFAM:PF07714) domain followed by seven WD-40 repeats (PFAM:PF00400), which presumably would fold into the characteristic propeller structure (41).

7. Very low expression and paucity of 6mA across retrotransposon sequences. Despite their abundance, retrotransposon loci in the *Loxodes magnus* MAC had little 6mA (0.04% of ApT vs. 10.9% of ApT in all predicted genes), which is likely due to their extremely low transcription, as observed for *Blepharisma* retrotransposons (42, 43). Genes with reverse transcriptase (PF00078) and endonuclease (PF14529) domains had a median expression of 0 transcripts per million (TPM); mean TPMs were ranked 11th and 13th lowest among 2767 Pfam domains.

8. Misannotation of DNA transposon families by RepeatClassifier. In comparison to the repeat families annotated as LINEs, those classified as DNA transposons or helitrons (Figure 4B, Table S2) were probably spurious annotations. A putative DNA/Zisupton family did not contain coding sequences with the expected SWIM zinc finger domain (PF04434) (44), whereas putative Unknown/Helitron-2 repeats encoded mostly WD-40 repeat sequences but not the endonuclease or helicase domains characteristic and necessary for helitron replication (45); RepeatClassifier similarly misclassified abundant WD-40 repeats in *Blepharisma* (42).

9. Homologs to ISXO2-like transposase in *Loxodes magnus*. Two proteins were annotated as "ISXO2-like transposase" (PF12762, DDE_Tnp_IS1595) in *L. magnus* MAC, however these do not appear to be contained in intact mobile elements, unlike in the hypotrich ciliates, where multiple DDE_Tnp_IS1595 transposase genes were found in the MAC genomes of *Oxytricha* (25) and *Stylonychia*

(46), and hundreds of insertion sequences (ISs) associated with these transposases were found in the *Oxytricha* MIC genome (47). Both the MAC- and MIC-encoded *Oxytricha* DDE_Tnp_IS1595 domain-containing genes are also substantially upregulated during development (25, 47). The *L. magnus* DDE_Tnp_IS1595-containing proteins had best BLASTP matches to protein sequences from *Blepharisma* and *Stentor* MAC genomes in GenBank's nr database. This suggested that these proteins may be domesticated transposases acquired before the hetrotrich/karyorelict divergence. *Blepharisma* genes containing the DDE_Tnp_IS1595 domain were upregulated during development like other transposase genes (43). As judged from a multiple sequence alignment of the *Loxodes* sequences of the seed alignment for PF12762 (using MAFFT v7.450 with E-INS-i algorithm) and inspection of IS1595 family alignments from TnCentral (48), one of the two sequences appeared to have a complete DDE motif, while the other has DEE. However, there are no signs of IS repeats identified by RepeatMasker flanking these genes in *L. magnus*. Thus it appears that the DDE_Tnp_IS1595 transposase genes in *Loxodes*/*Blepharisma*/*Stentor* may have been acquired independently from those in *Oxytricha* and may no longer be involved in transposition.

10. Cluster of mobile element genes horizontally transferred from *Rickettsia* bacteria to *Loxodes magnus*. Few conserved domains related to DNA transposons were detected in the *L. magnus* predicted proteomes. A phage integrase family domain (Pfam PF00589) was found in 28 predicted genes in the MAC genome, but none were associated with interspersed repeats. A YhG-like transposase domain (PF04654) was also found in one predicted protein. This predicted protein encodes an additional domain, "RecT" (PF03837), annotated as DNA-binding, on its N-terminus. BLASTP to GenBank's nr database revealed top hits to both domains, from different *Rickettsia* proteins (E-values of 7e-115 and 1e-81 for PF04654 and PF03837, respectively). The gene encoding this protein (000111F.g160) had sense-strand 6mA characteristic of *L. magnus* DNA (see main text, "*Loxodes* MACs have characteristics of both active chromatin and heterochromatin"), so this gene is probably not from residual contaminating DNA from *Rickettsia* that might have been co-cultured with *L. magnus*. A gene close by (000111F.g16) encodes an integrase (PF13683, "Integrase core domain"). The protein encoded by this gene differs from the phage integrase domain proteins, which have best BLAST matches to *Woesearchaeota*. Additional BLAST searches revealed an ~9.6 kbp cluster of six predicted genes (000111F.g159-g164) that all had best matches to *Rickettsia*, so it is likely all were horizontally transferred to *Loxodes* at once.

Since integrases insert DNA, rather than remove and insert it like transposases (49), it seems unlikely that any of the proteins with integrase domains is involved in editing out pieces of *Loxodes* genomes.

11. Histone H3 homologs in *Loxodes magnus*. The *L. magnus* genome encodes multiple histone H3 homologs which cluster into three groups, one among canonical H3 and H3.3 (cluster 1), a divergent group (cluster 2), and one nested among centromeric H3 (cenH3) sequences (cluster 3) (Figure S12). The histone H3 antibody used was raised against an immunogen with 91.7% identity to cluster 1, but only 75% and 50% to clusters 2 and 3 respectively (Abcam Scientific Support, pers. comm.). Similarly the immunogen for the anti-H3K4me3 antibody had 100% identity to cluster 1 but <65% for clusters 2 and 3, and immunogens for the anti-H3K9ac and H3K9me3 antibodies only matched cluster 1.

12. Homologs of 6mA methyltransferases in *Loxodes magnus*. Based on BLASTP and TBLASTN searches in the *Loxodes magnus* MAC proteome and genome, respectively, we were unable to find convincing homologs of DAMT1 (METTL4), the 6mA methyltransferase originally characterized in *Caenorhabditis elegans* (50, 50, 51). We did detect four homologs of the *Tetrahymena* methyltransferase subunits MTA1 (AMT1) and MTA9-B/MTA9 (AMT6/7) (51, 52) using BLASTP (E-values 6e-22 to 6e-43; sequences available at https://doi.org/10.17617/3.BOFMWS). Using the *Loxodes* sequences as BLASTP queries vs. *Tetrahymena* predicted proteins, the best matches of two of these proteins are METTL3 (AMT4) and METTL14 (AMT3), which are distantly related paralogs in the same family as AMT1 and AMT6/7, that are considered to form a heterodimer responsible for m6A in RNA, rather than 6mA in DNA (53) The other two sequences had best BLASTP matches to a *Tetrahymena* methyltransferase known as AMT2 or TAMT that is related to the other AMTs (51, 54). As judged by InterPro searches, the two *Loxodes* proteins also have the C-terminal zinc fingers characteristic of AMT2/5, unlike AMT1 and AMT6/7 (51).

Aside from the catalytic methyltransferase subunits, there were no convincing matches (E-value < 1e-3) to *Tetrahymena*'s p1 and p2 proteins (also known as AMTP1 and AMTP2), that form a complex together

with MTA1/MTA9 (51, 52, 55, 55) in *L. magnus* predicted MAC and MIC proteins with BLASTP, whereas there were convincing matches in predicted proteins from the *Blepharisma stoltei* ATCC30299 MAC genome (E-value 5e-45 and 4e-16, respectively). As judged by a BLASTP search, *Blepharisma stoltei* also has likely orthologs of both AMT1 and AMT2.

Though there are contradictory reports on the exact role of AMT2 in *Tetrahymena* 6mA deposition (51, 54), it is currently thought that AMT2 is responsible for asymmetric 6mA (hemi-methylation) and AMT1 for symmetric 6mA (full methylation) (56). *Blepharisma stoltei* which has full methylation has likely orthologs of both AMT1 and AMT2, which is consistent with our observation of full 6mA genomic methylation in its genome. In contrast, it appears that *Loxodes* has only retained proteins for 6mA hemi-methylation and lost the entire full methylation complex (AMT1, AMT6/7, AMTP1 and AMTP2).

13. Unsuccessful searches for telomerase RNA (TR), telomerase protein (TERT) and telomere binding proteins. We failed to detect telomerase RNA (TR) in *Loxodes* and in the heterotrichs *Stentor coeruleus* and *Blepharisma stoltei* using Infernal searches with all available TR models from RFAM. RFAM has separate models for TRs from ciliates (RF00025), vertebrates (RF00024), and fungi (RF01050 and RF02462), which indicates that TRs diverge much more than other ncRNAs like tRNAs and rRNAs, and so may be difficult to detect with Infernal alone outside of the ciliate classes used to create the seed alignment underlying RF00025.

In *Loxodes*, we were also unable to detect homologs of telomerase protein (TERT) via HMMER3 searches of the TERT-specific PFAM domain PF12009 (via InterProScan). In the heterotrichs, we found TERT homologs in the MAC genome of *Stentor coeruleus*, but not in that of *Blepharisma stoltei*. BLASTP and TBLASTN searches with the *Stentor* TERT proteins as queries vs *Loxodes* proteins and genomes also did not reveal compelling homologs (no hits with E-value < 1e-3).

An independent research group used a different method that detects TR together with its type 3 promoter, and found candidate TRs in the heterotrich ciliates *Stentor coeruleus* and *Condylostoma magnum* (57), but these still need experimental verification. Unfortunately, such searches require knowledge of the telomeric repeat, which we lacked.

We also checked for telomere binding proteins (TEBPs) which, from our experience diverge rapidly, but again did not detect such proteins. Multiple homologs of TEBPs with the PFAM POT1 domain (PF02765) are present in *Stentor coeruleus* and *Blepharisma stoltei* (for *Stentor* see, e.g. UniProt ID: A0A1R2BA73).

Drosophila presents a well-known case of telomeres that are not based on repeats synthesized by TERT and TR, but rather on non-LTR retrotransposons (58). If TERT, TR and TEBPs are indeed all absent in *Loxodes*, an alternative form of telomerase-independent telomere maintenance may have evolved.

SI Discussion

Plausibility of previously reported differential genome amplification and IES excision in *Loxodes*. A previous study reported that the copy-number of protein coding genes in an uncultivated *Loxodes* sp. varied across at least four orders of magnitude (59). This conclusion was based on qPCR quantification of four genes–actin, RS11, EF-1a, and alpha-tubulin–using single-cell multiple-displacement amplification (MDA) products as templates and the SSU rRNA gene as the common reference across samples.

To take the results at face value, we have to assume that: (a) there is little amplification bias from whole genome sequencing, (b) all the genes targeted (except SSU rRNA) are single-copy in the haploid genome, (c) PCR primers are specific despite being degenerate and do not amplify any paralogs or non-target sequences, and (d) the four protein-coding genes fortuitously represent the full dynamic range of copy-number variation after differential amplification.

The previous study used multiple-displacement amplification (MDA) products from single cells as templates for qPCR. MDA itself is known to amplify DNA unevenly with pronounced amplification biases (point a above). Furthermore, the genes whose copy number they quantified by qPCR are not single-copy in *Loxodes magnus*, but have multiple paralogs or copies, e.g. > 80 copies of actin in both the MAC and MIC genomes. The degenerate primers used for qPCR would not be specific enough to distinguish between paralogs (points b, c).

Even if we accept that there is differential amplification in the somatic genome (point d), the degree of copy number variation claimed in the prior study is quantitatively incompatible with the relative DNA content observed in *Loxodes* MACs vs. MICs. The DNA content of karyorelict MACs is "paradiploid", meaning that it is on the same order of magnitude as the diploid zygotic nucleus precursor (27, 60). Therefore, if there is amplification of specific loci in the MAC, there must be a corresponding similar amount of DNA that is eliminated during development, such that the total amount is about the same. For example, if 10% of the genome is amplified 10-fold, then the remaining 90% must be eliminated.

However, the previous study reported relative abundances spanning 4 orders of magnitude. If we assume that the gene with the lowest abundance is single-copy (i.e. no amplification), that means some loci are amplified 10^4-fold compared to the zygotic nucleus. To keep the total DNA quantity the same, loci that are amplified 10^4-fold can constitute a maximum of 10^{-4} (0.01%) of the genome, in which case all other DNA must be eliminated.

Even if loci with 10^4-fold amplification are outliers, and the mean amplification lies between 10- to 100-fold, this still implies that 90% to 99% of the MIC DNA must be eliminated. While on the higher end, this may be consistent with the degree of elimination known from other ciliates, e.g. *Paramecium caudatum* with 1.3 Gbp MIC vs. 30 Mbp MAC genome, i.e. 98% eliminated (61). Such extensive elimination, however, would be easily detected by differential k-mer abundances in MIC vs. MAC libraries, even if sort purity was low, but we did not observe this.

We therefore judge that differential genome amplification in *Loxodes* to the extent claimed (up to 10^4-fold) is unlikely. The variability in copy number previously reported is thus a consequence of insufficient knowledge of the underlying genome architecture and gene organization at the time, and may have been compounded by insufficient primer specificity and amplification biases from MDA.

The previous study further concluded that *Loxodes* has IESs by mapping transcriptome data (as a proxy for somatic MAC sequence) to single-cell genome assemblies from MDA products (as a proxy for MIC sequence). They reasoned that because the polymerase in MDA has a bias for amplifying longer DNA molecules, it should preferentially amplify MIC DNA because it is less fragmented than MAC, based on results of a prior study on *Chilodonella uncinata* (62). *Chilodonella* has extensive genome fragmentation with very short (~1 kbp) MAC nanochromosomes, so this assumption may apply to that ciliate, but there is no evidence from their study or ours that *Loxodes* or other karyorelicts have nanochromosomes. So the applicability of MDA to enrich MIC DNA in *Loxodes* is questionable, and also contradicts the use of MDA products as templates to estimate gene copy number in the MAC.

Furthermore, the previous study also found indels present in the MDA assembly but not the transcriptome; these indels were bound by terminal direct repeats (TDRs), which they took to be a diagnostic feature of IESs in general. However, there was no other evidence that these are actually

localized to the MIC. In addition to amplification biases mentioned above, MDA is also known to produce chimeric sequence artifacts (63) and hence is not suitable for evaluating genome editing without other supporting evidence. In this study we have observed monoallelic indel polymorphisms bound by TDRs, which probably originate from mobile elements, so we propose that the putative IESs from the previous study were in fact such monoallelic indels.

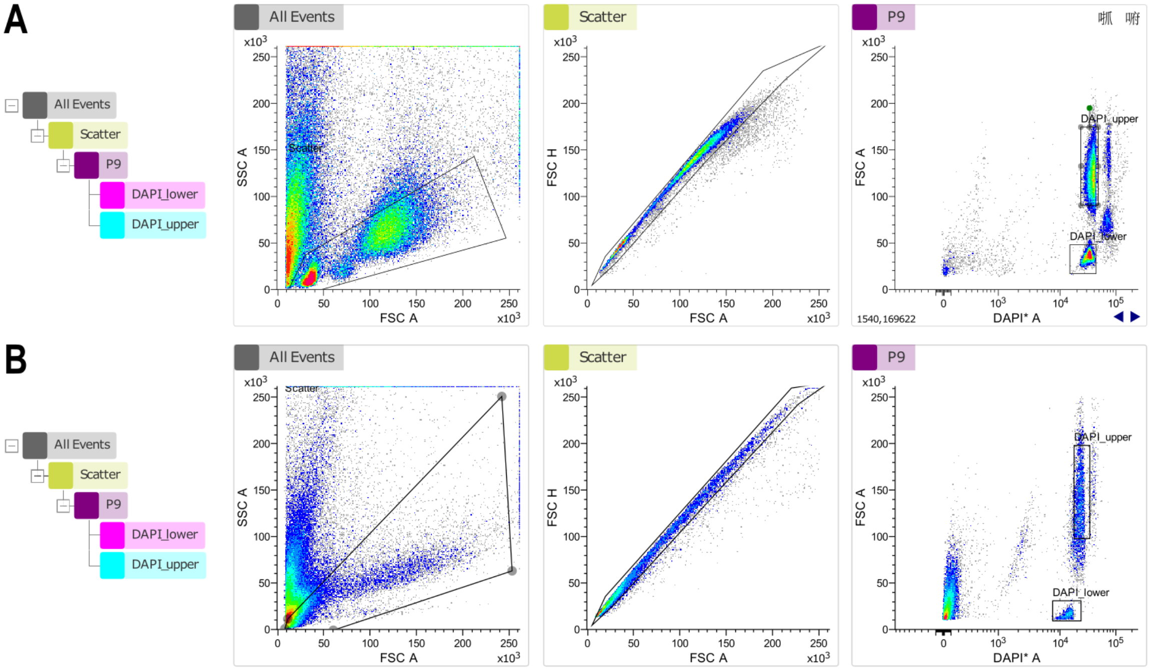

Fig. S1. Gating scheme and scatter plots for fluorescence-activated sorting of *Loxodes* nuclei. (A) *Loxodes magnus*, (B) *Loxodes striatus* (representative runs, 100,000 events depicted per plot).

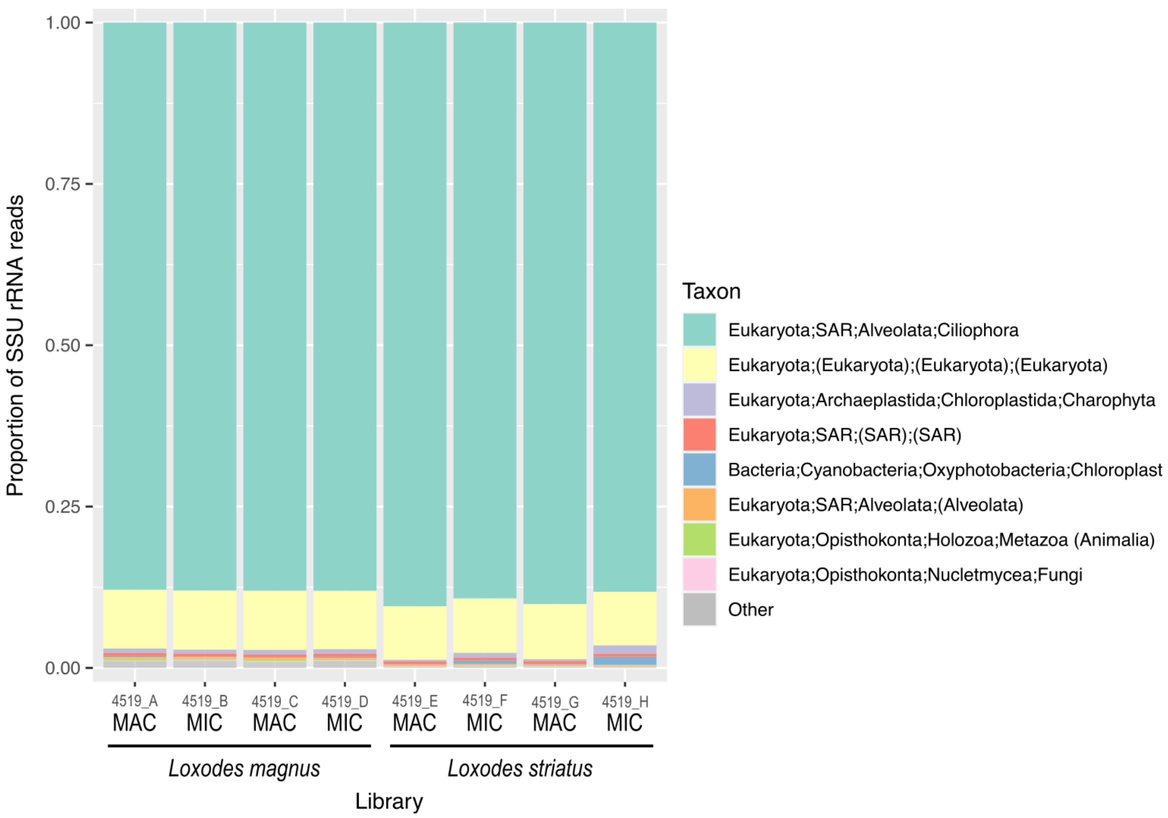

Fig. S2. phyloFlash taxonomic summaries for genomic Illumina libraries of sorted nuclei. Taxon names in parentheses represent sequences that could not be classified to that rank, the lowest named level was used instead.

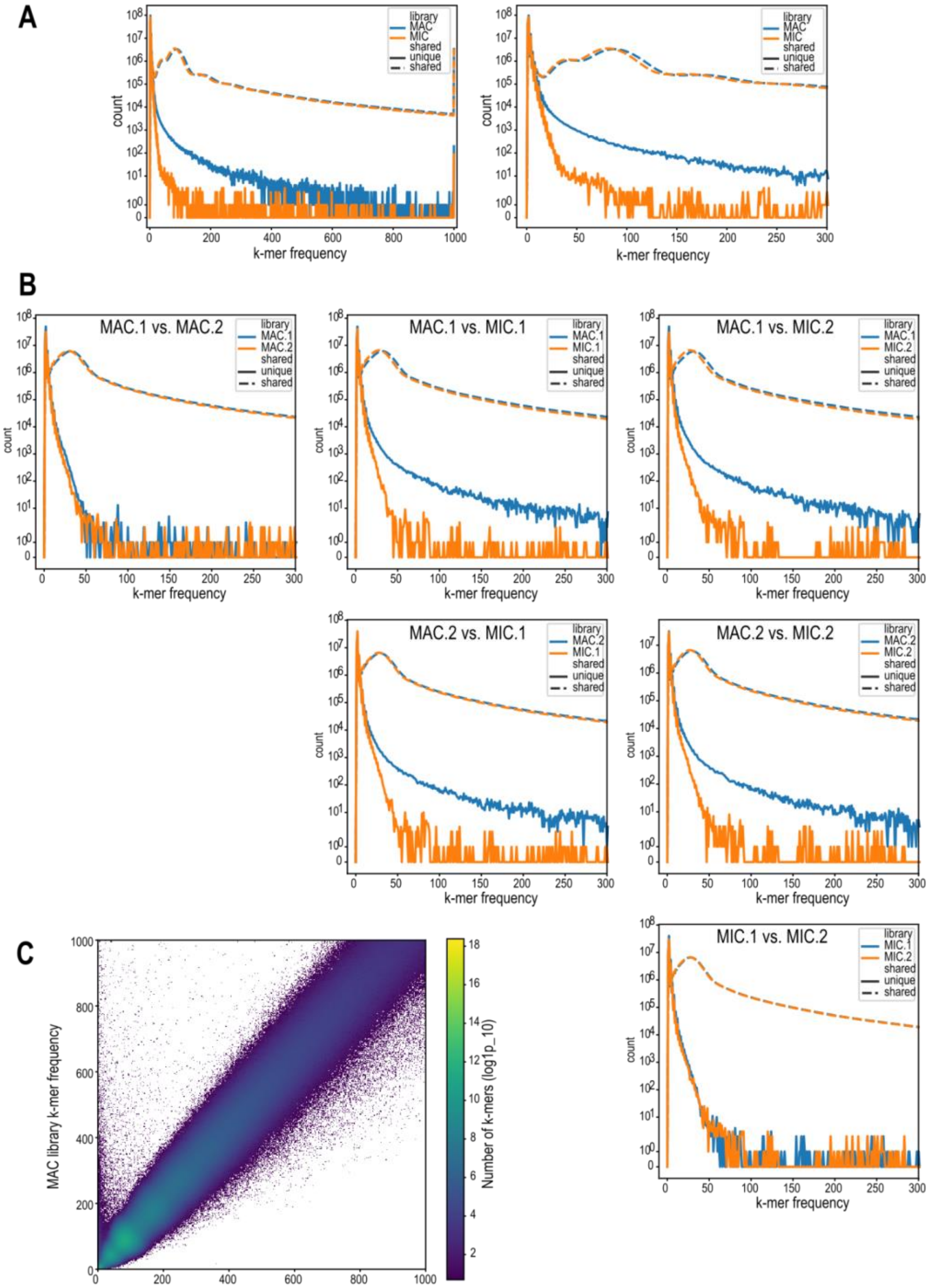

Fig. S3. k-mer comparison of Loxodes magnus MIC vs. MAC libraries. (**A**) k-mer multiplicity plot (right: detail) for shared (dashed lines) vs. unique (solid lines) 21-mers in pooled MAC (blue) vs. MIC (orange) sequence libraries. (**B**) k-mer multiplicity plots for shared vs. unique k-mers, pairwise comparison of individual replicates of MAC and MIC, subsamples of 150 M reads per library; x-axis truncated at 300× frequency. (**C**) Heatmap comparing frequency of 21-mers in MIC vs. MAC libraries, axes to 1000× frequency.

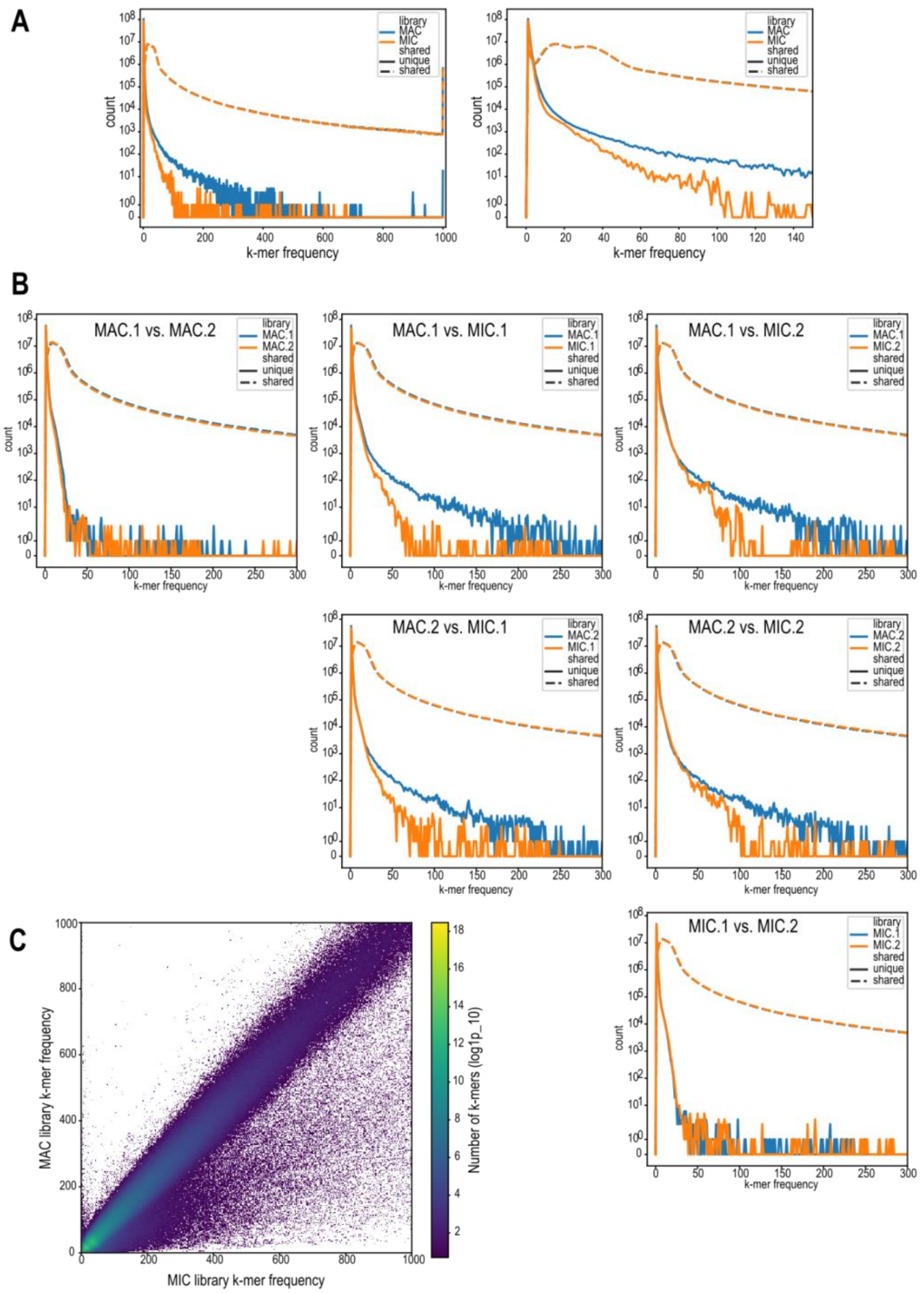

Fig. S4. k-mer comparison of Loxodes striatus MIC and MAC genomic libraries. Panel captions as for Fig. S3.

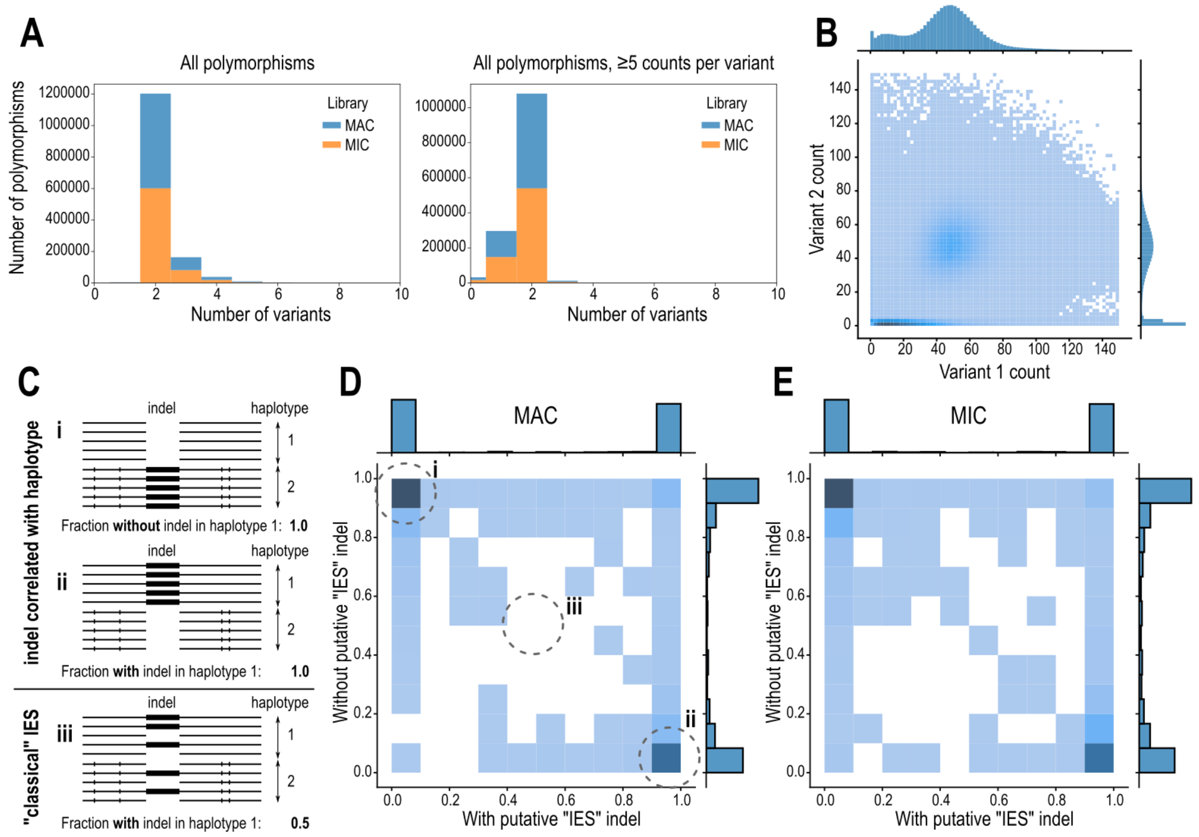

Fig. S5. Single nucleotide polymorphisms (SNPs) in *Loxodes magnus* and correlation with indels. (**A**) Number of variants per SNP (naive variant counting), before and after low-abundance variants observed ≤5 times were removed. (**B**) Counts of variant 1 vs. variant 2 per SNP (both MIC and MAC libraries, combined); ratio is ~1:1 on average; SNPs where variant 2 has counts ≤5 are likely to be sequencing or variant-calling errors. (**C**) Diagram of how putative "IES" indels were counted relative to haplotagged reads. Individual HiFi reads were tagged as haplotype 1 or 2 based on SNPs relative to the reference assembly. For each set of reads with or without a given "IES" insert, the numbers of reads per haplotype were counted. (**D**, **E**) 2-D histograms (linear color intensity scale) of the fraction of reads assigned to reference haplotype for reads with insert (horizontal axis) vs. without insert (vertical axis), for each putative "IES" indel position, for MAC vs. MIC libraries (D, E respectively). In most cases, presence of insert was correlated with the haplotype, as expected if they were monoallelic indel polymorphisms (panel C scenarios i and ii), whereas classical IESs that have reached fixation would be expected to be independent of haplotype (i.e. in the center of the heatmap, panel C scenario iii).

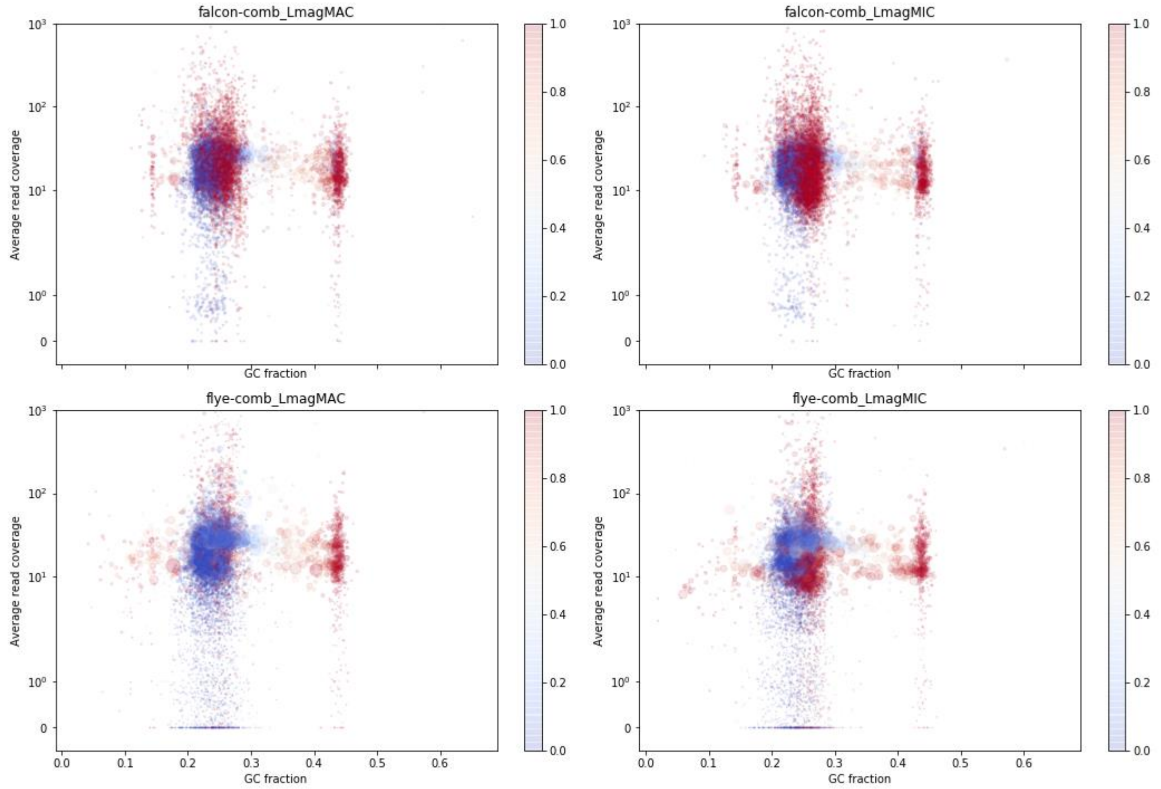

Fig. S6. Blob plots (read coverage vs. GC% per contig) for *Loxodes magnus* long-read genome assemblies. Plot symbol areas are scaled proportionally to contig length; color scale represents fraction of contig covered by low-complexity tandem repeats.

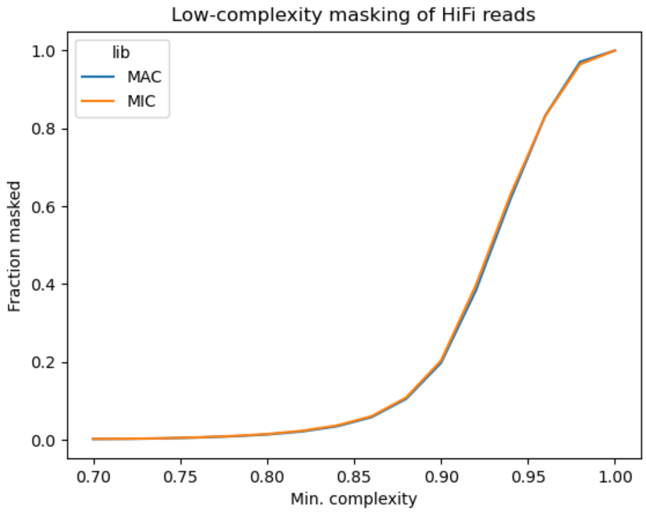

Fig. S7. Fraction of bases masked vs. minimum sequence complexity cutoff in masking of low-complexity sequence in *Loxodes magnus* HiFi reads. Fractions masked at each cutoff level are similar for MICs and MACs, implying that both types of nuclei have similar amounts of low-complexity sequence content.

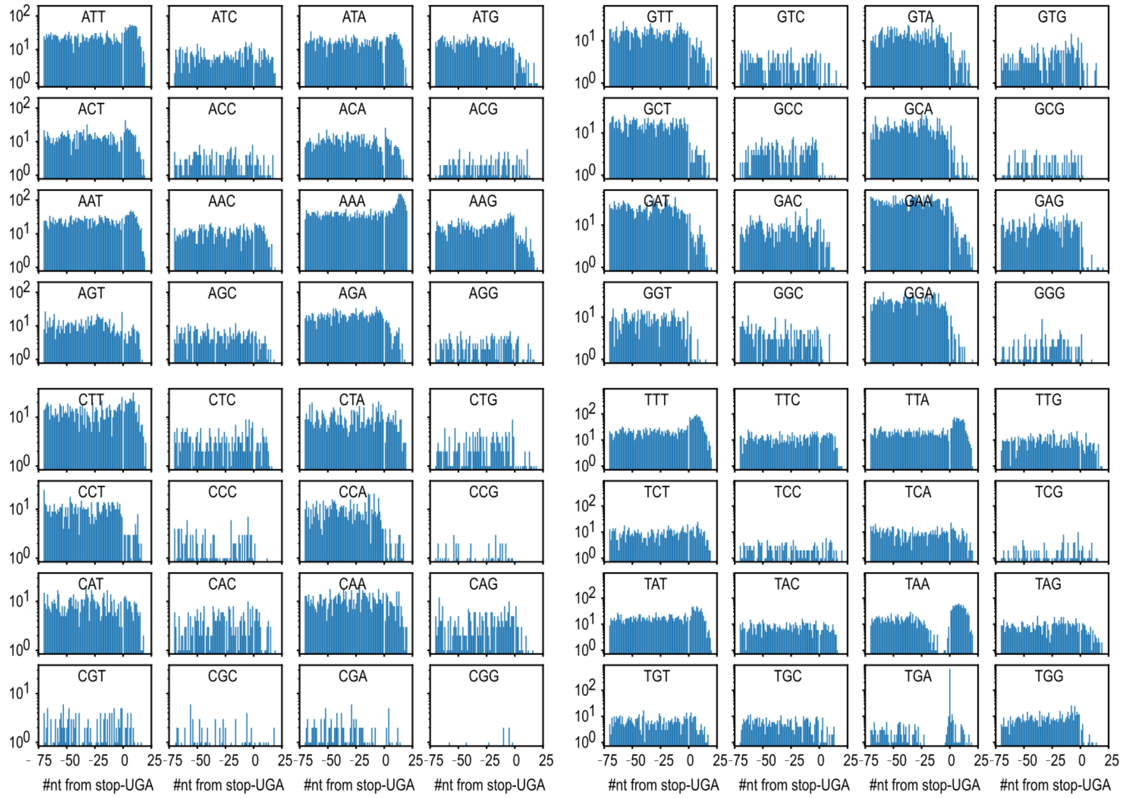

Fig. S8. Counts of codons relative to predicted stop-UGAs in *Loxodes magnus* transcripts.

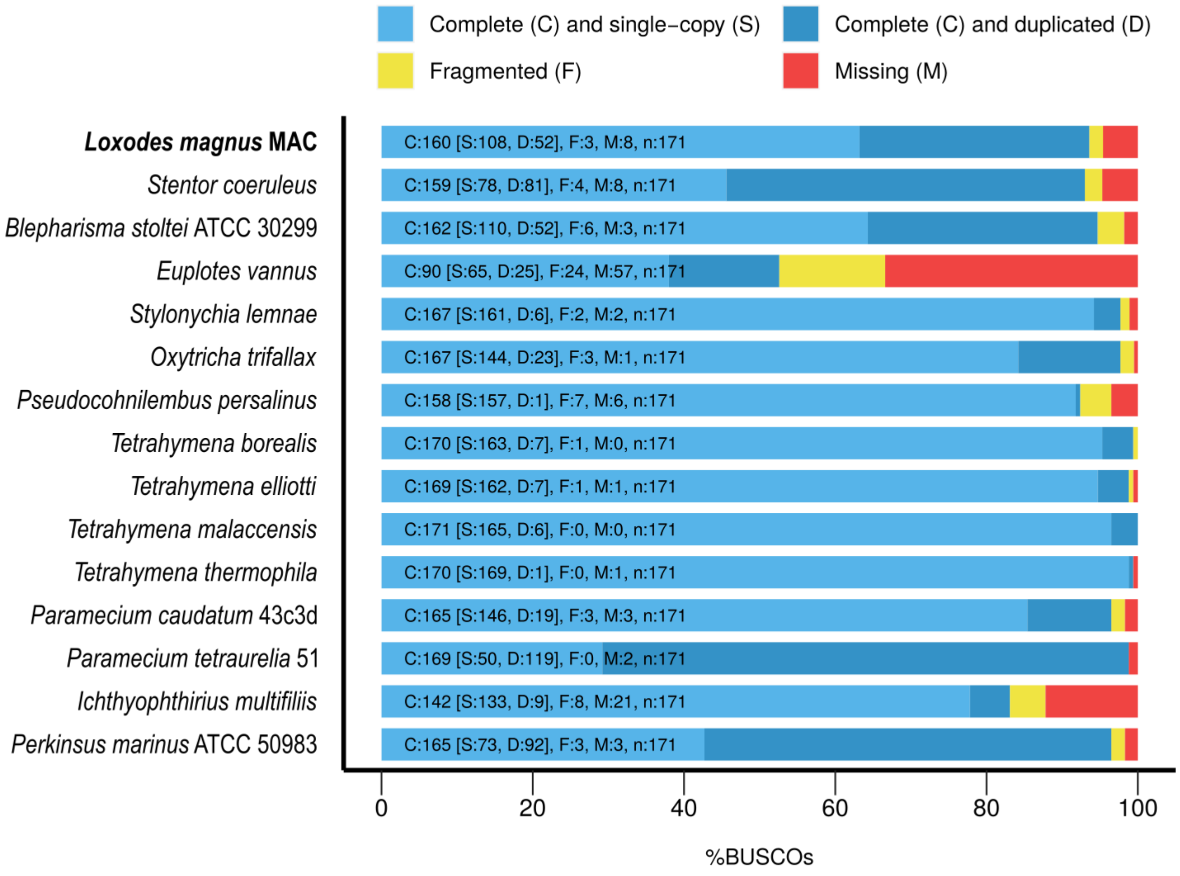

Fig. S9. Genome completeness scores using BUSCO conserved orthologs. Scores calculated using the BUSCO Alveolata marker set, including select ciliates like *Tetrahymena thermophila*. Shown are ciliate MAC genomes (predicted proteins in published annotations) with *Perkinsus marinus* as a relevant non-ciliate, alveolate outgroup.

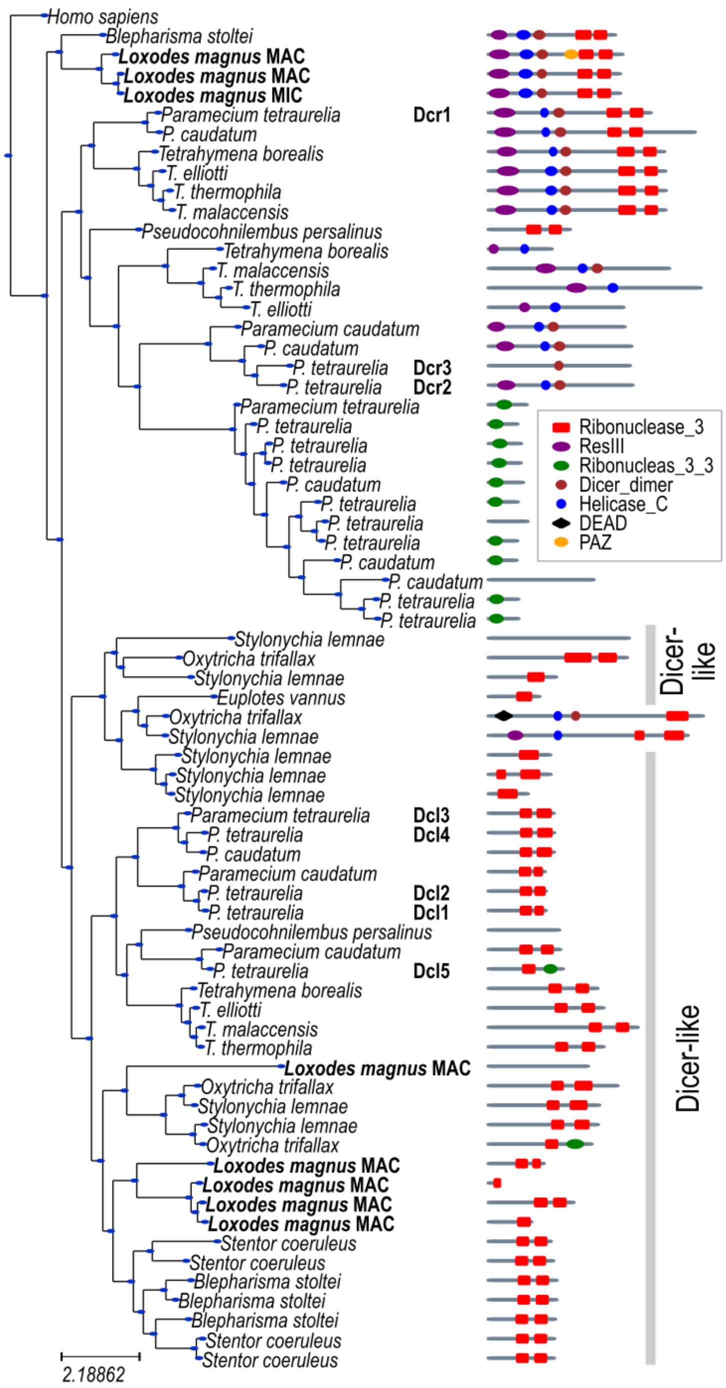

Fig. S10. Phylogenetic tree of Dicer or Dicer-like domains (RNase III domain-like superfamily SSF69065) in ciliate genomes. Tree shown alongside Pfam domain annotations in the corresponding proteins. Named Dicer (Dcr) or Dicer-like (Dcl) genes in *Paramecium tetraurelia* are indicated.

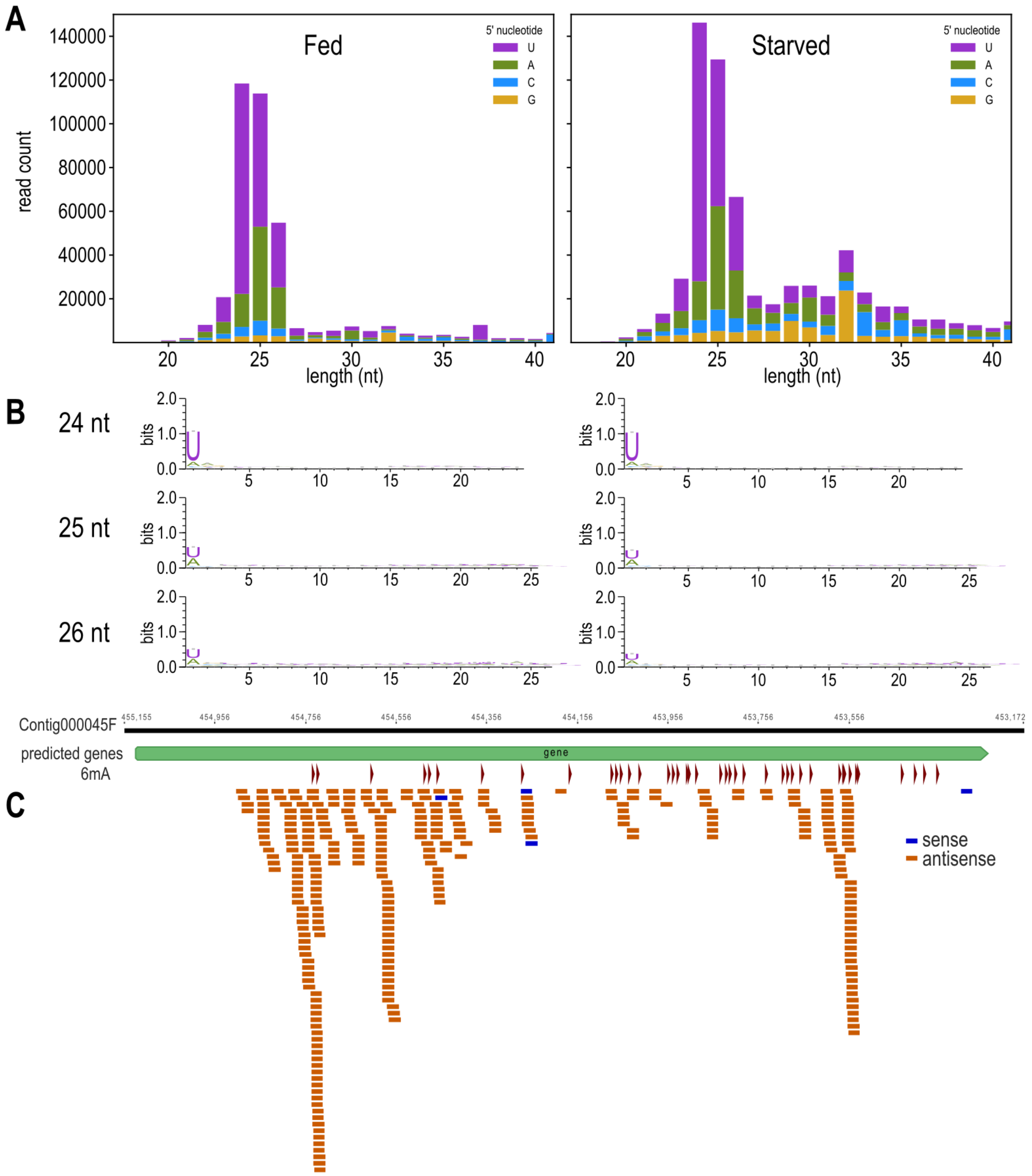

Fig. S11. Characteristics of *Loxodes magnus* sRNAs. (**A**) Length distributions of mapped sRNAs from fed (left) vs. starved (right) populations of *Loxodes magnus*. Bar colors: 5’ nucleotide base. (**B**) Sequence logos of 24, 25, and 26 nt sRNAs from fed (left) vs. starved (right) *L. magnus*. (**C**) Mapping of 24 nt sRNAs to a representative *L. magnus* gene, showing strand-bias in both the sRNA (colored bars) and the 6mA modifications (red arrowheads).

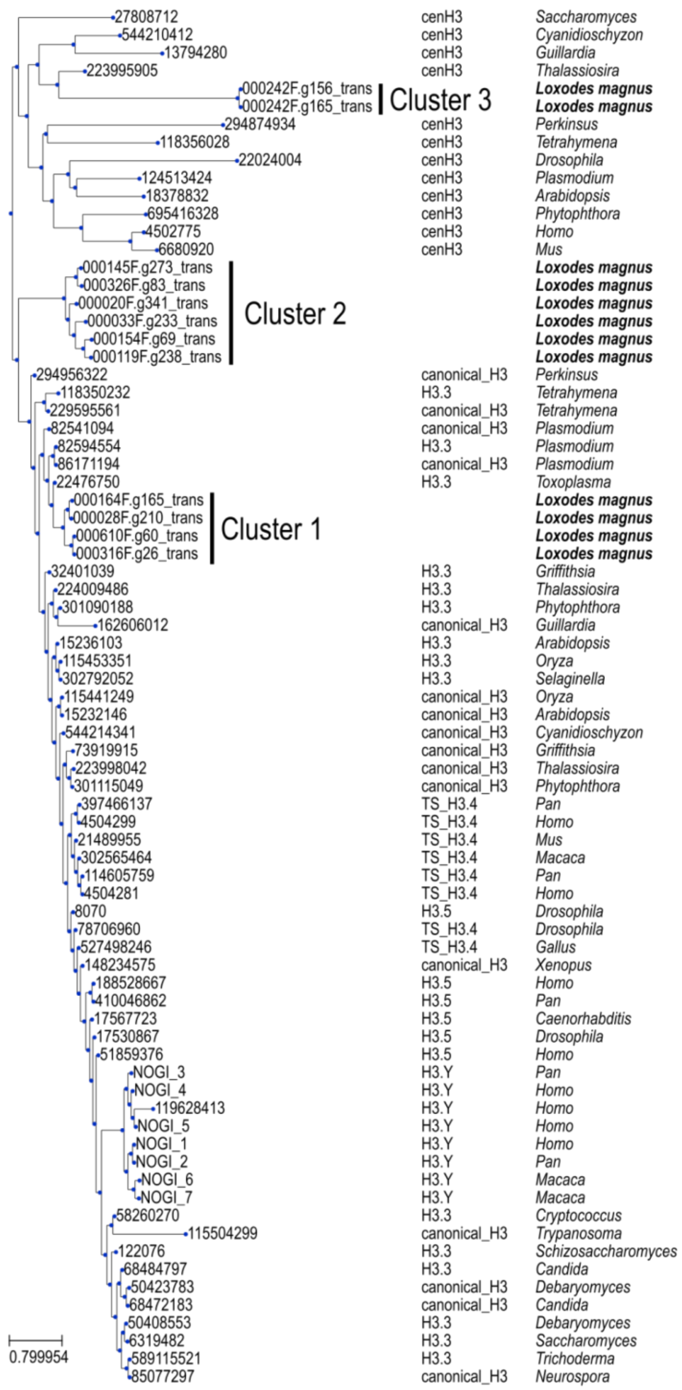

Fig. S12. Phylogenetic tree of histone H3 sequences from model organisms and *Loxodes magnus*. Reference sequences and histone variant classification are from HistoneDB 2.0. Histone H3 sequences from *L. magnus* fall into three clusters (indicated). Scale bar: Substitutions per site.

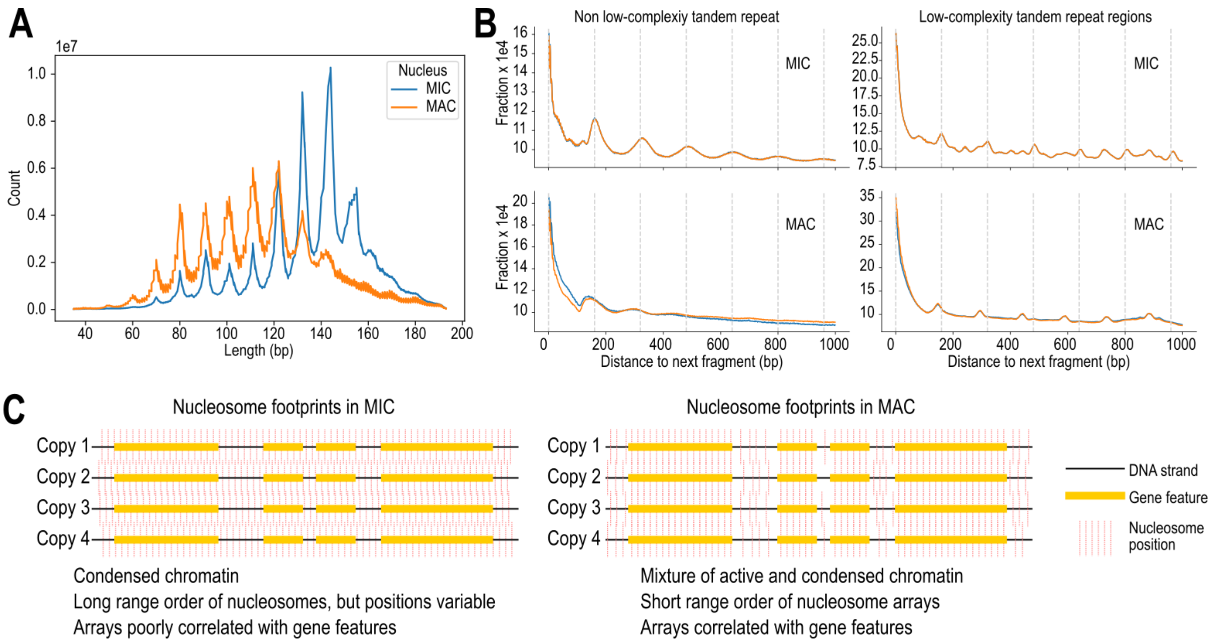

Fig. S13. Properties of *Loxodes* nucleosomes. (**A**) Fragment size distribution of dsDNase digest nucleosomal DNA libraries for MIC (blue) vs. MAC (orange), from merged paired-end reads. (**B**) Comparison of global phaseograms of nucleosomal DNA libraries when low complexity repeat regions are masked as in Figure 5D (left), vs. for low complexity repeat regions only (right). (**C**) Schematic model of nucleosome arrays in *Loxodes* MIC vs. MAC nuclei.

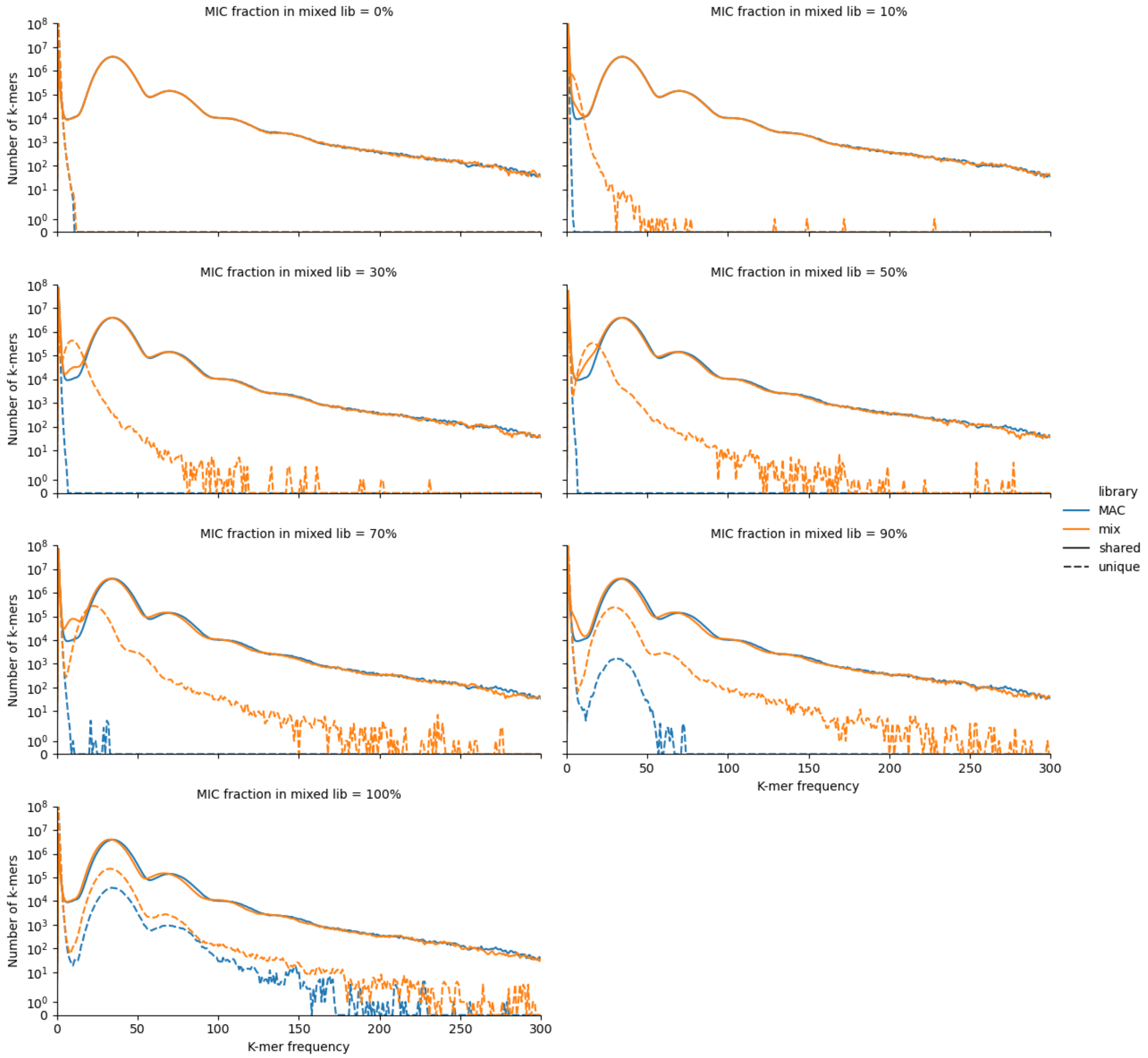

Fig. S14. k-mer comparisons (k=19) of simulated pure MAC library vs. MAC+MIC mixture. Simulations with increasing percentage of MIC ("mix"). Solid lines – shared k-mers, dashed lines – unique k-mers, blue – pure MAC library, orange – mixed library.

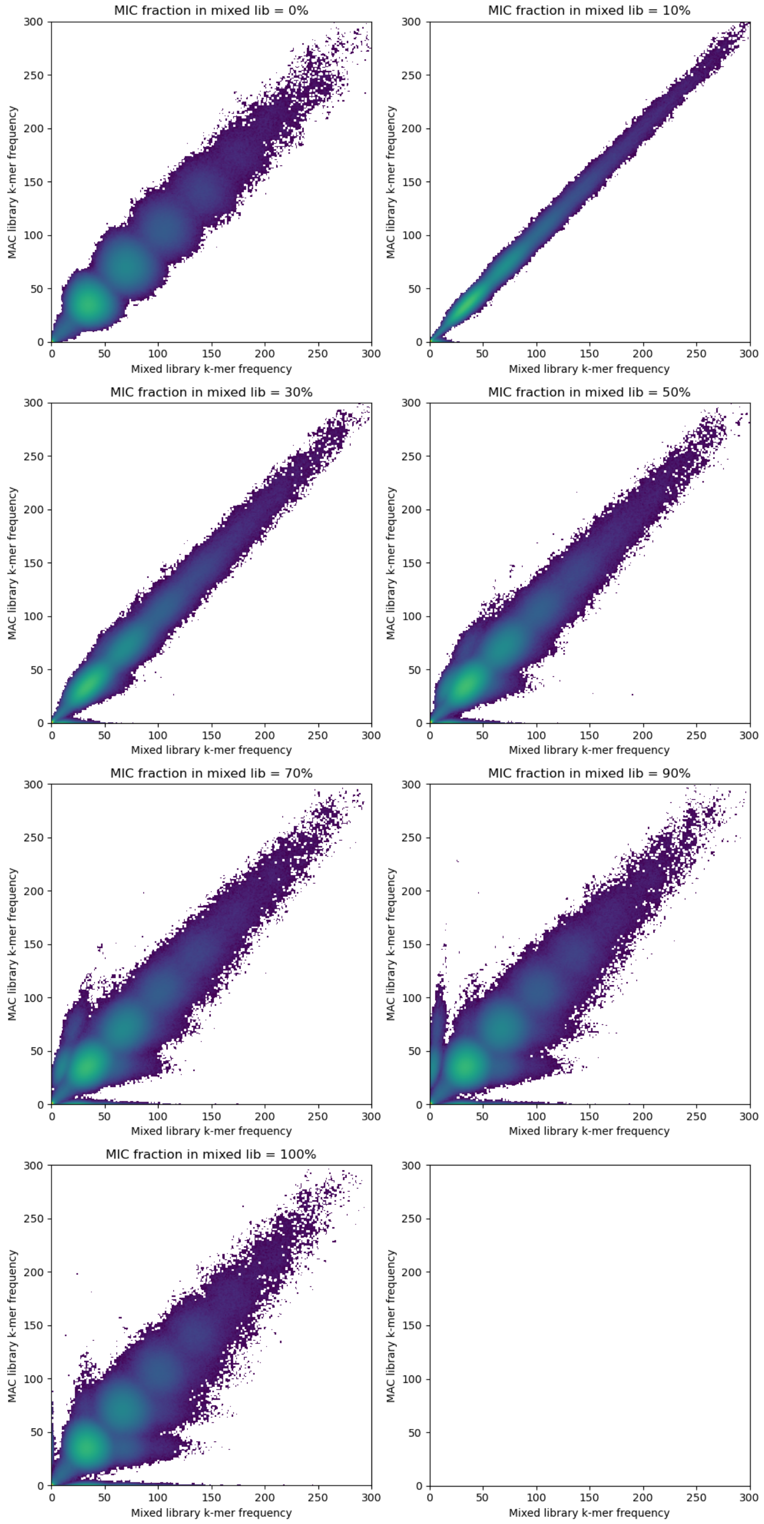

Fig. S15. Heatmaps of k-mer frequency in comparisons of simulated pure MAC library (vertical axis) vs. MAC+MIC mixture. Ordered by increasing percentage of MIC (horizontal axis); color scale intensity represents number of k-mers.

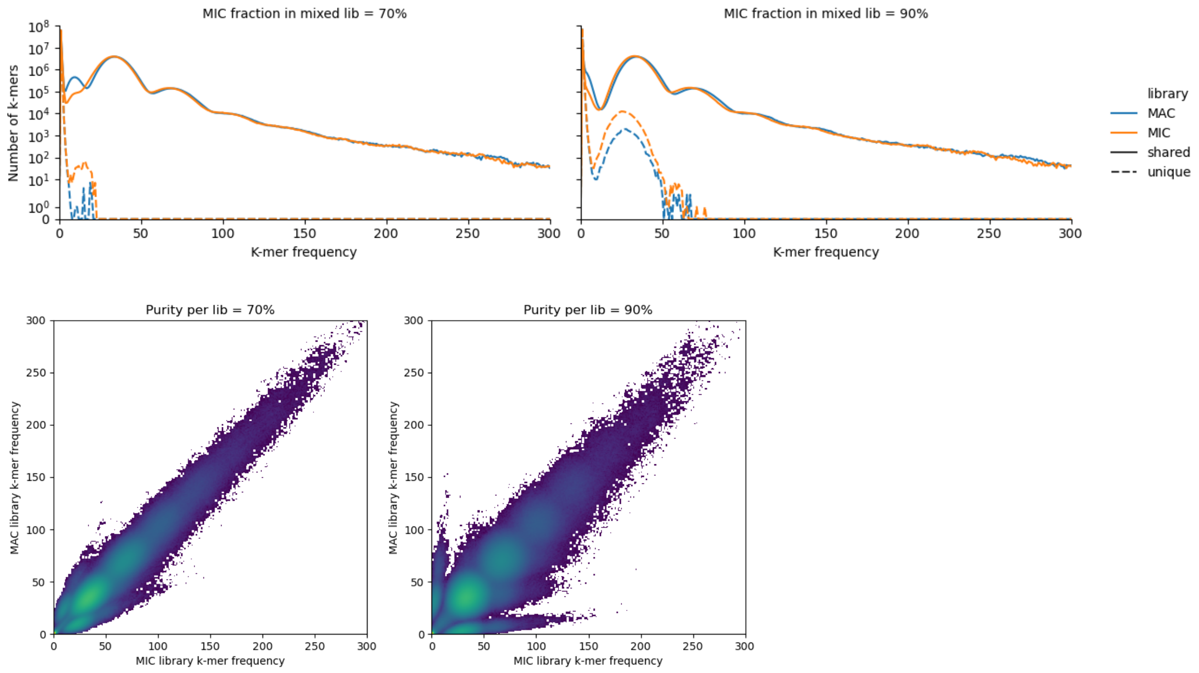

Fig. S16. Simulated k-mer contamination. (**A**) K-mer comparisons (k=19) of simulated MAC vs. MIC libraries, each with cross-contamination from the other sequence type. Left: both libraries 70% pure, right: both libraries 90% pure. (**B**) Heatmaps of k-mer frequency in comparisons of simulated cross-contaminated MAC vs. MIC libraries.

Table S1. Genome assembly metrics for different assemblers and sorted nuclei libraries for *Loxodes magnus*. Low complexity regions were merged if overlapping, and only regions >1 kbp were counted. Interspersed repeat total length: after overlapping annotations were merged. ND – not determined.

**Assembler**	**Falcon**	**Flye**	**MEGAhit**	**SPAdes**	**Falcon**	**Flye**	**MEGAhit**	**SPAdes**
**Nucleus**	**MAC**	**MAC**	**MAC**	**MAC**	**MIC**	**MIC**	**MIC**	**MIC**
Library type	PacBio HiFi	PacBio HiFi	Illumina 2×150 bp	Illumina 2×150 bp	PacBio HiFi	PacBio HiFi	Illumina 2×150 bp	Illumina 2×150 bp
Number contigs	7858	8222	619582	3287736	9387	9231	602227	3459997
Total length (Mbp)	706	626	404	793	848	805	399	815
Largest contig (Mbp)	2.89	2.77	0.392	0.450	2.867	2.98	0.509	0.502
GC (%)	26.2	25.91	24.65	24.45	26.1	25.89	24.65	24.48
N50 (kbp)	177	204	10.2	6.59	193	244	10.3	6.57
N75 (kbp)	65.4	75.8	2.34	1.61	68.6	93.4	2.33	1.60
L50	802	647	3772	6997	922	749	3706	6983
L75	2476	1954	17488	30669	2809	2116	17420	30757
# N's per 100 kbp	0	3.43	0	70.94	0	3.68	0	63.4
Low complexity (Mbp)	231	172	ND	ND	359	344	ND	ND
Interspersed repeats (Mbp)	454	ND	ND	ND	571	ND	ND	ND
Non-repetitive (Mbp)	229	ND	ND	ND	245	ND	ND	ND

Table S2. Summary of RepeatMasker annotations in *Loxodes magnus* MAC vs. MIC genome assemblies for interspersed repeat families found by RepeatModeler with a classification to known mobile element class, out of a total of 170 putative repeat families. Full length copies are defined as annotations >80% and <120% of consensus length.

Repeat family	Classification	Cons. length (bp)	Total annotated length (bp)		No. copies		No. full length copies	
			MAC	MIC	MAC	MIC	MAC	MIC
rnd-1_family-2	Unknown/Helitron-2	3032	64725198	61022801	2777	2805	164	163
rnd-6_family-790	Unknown/Helitron-2	13725	5659472	5410868	2623	2564	57	63
rnd-1_family-27	LINE/RTE-X	6042	4861662	4818431	2816	2870	311	294
rnd-1_family-19	LINE/RTE-BovB	5380	4183278	4405536	3132	3317	313	339
rnd-1_family-63	LINE	5979	3393684	3384063	3325	3402	138	135
rnd-1_family-12	LINE/RTE-X	5327	3236685	3462892	3644	3928	74	74
rnd-1_family-8	Unknown/Helitron-2	6643	2946027	2767078	1412	1375	83	72
rnd-1_family-60	LINE/RTE-X	5950	2784869	2675233	1765	1777	159	151
rnd-1_family-18	LINE/RTE-BovB	3770	2440970	2536625	5610	5844	77	84
rnd-1_family-5	Unknown/Helitron-2	963	2315210	2136144	665	696	214	218
rnd-1_family-80	LINE/RTE-X	3159	2099005	2276340	1804	1924	318	335
rnd-4_family-203	Unknown/Helitron-2	7805	2052133	2058648	1794	1829	48	46
rnd-1_family-35	LINE/RTE-X	3420	1986760	2079082	1720	1825	224	237
rnd-1_family-87	LINE/RTE-BovB	4907	1519913	1749801	2380	2588	29	32
rnd-5_family-51	LINE/CR1-Zenon	3464	1082459	1154752	887	954	123	129
rnd-1_family-284	LINE	2171	913602	907091	1010	1007	227	217
rnd-1_family-9	Unknown/Helitron-2	2032	804242	867379	2753	2925	64	76
rnd-1_family-3	Unknown/Helitron-2	1055	767788	713631	791	797	400	394
rnd-5_family-124	LINE/Proto2	1101	658646	670670	1160	1186	350	357
rnd-1_family-409	LINE/CR1-Zenon	2825	657715	655773	460	483	133	116
rnd-1_family-98	Unknown/Helitron-2	1233	646506	704277	469	503	23	30
rnd-1_family-42	Unknown/Helitron-2	1768	571872	538255	425	432	48	44
rnd-1_family-395	LINE	1041	533381	569970	637	668	465	499
rnd-1_family-69	LINE/RTE-X	2717	500464	473997	373	372	121	118
rnd-6_family-684	Unknown/Helitron-2	4143	382572	364527	561	562	19	15
rnd-4_family-192	LINE/RTE-BovB	5540	285209	302764	1394	1473	10	9
rnd-6_family-221	LTR/Pao	5722	261010	381599	353	532	4	4
rnd-1_family-61	LINE	2131	257315	272059	507	517	2	1
rnd-1_family-112	LINE/RTE-X	2037	176947	183977	378	395	3	3
rnd-5_family-642	DNA/Zisupton	1851	161878	162381	164	165	34	37
rnd-1_family-125	LTR/Copia	281	152222	156034	655	675	431	441
rnd-1_family-154	LINE	1997	146969	134998	253	243	13	10
rnd-1_family-10	Unknown/Helitron-2	320	116490	67605	191	180	13	14
rnd-1_family-212	Unknown/Helitron-2	1188	66226	65841	132	142	8	6
rnd-1_family-94	Unknown/Helitron-2	546	38712	41004	131	137	10	8
rnd-1_family-133	LINE/RTE-X	888	22562	26696	56	61	1	2
rnd-1_family-436	LINE/RTE-RTE	445	20925	19592	65	67	35	30
rnd-1_family-123	LINE/R2	316	12516	14397	96	111	9	10

rnd-1_family-384	LTR/Pao	146	2889	2758	22	21	7	7
rnd-1_family-40	Unknown/Helitron-2	468	1395	1741	8	7	1	2
rnd-1_family-142	LINE	317	1306	1191	9	9	1	1

Table S3. Cell counts of starved vs. fed cultures of *Loxodes magnus* used for RNAseq of non-dividing vs. actively dividing populations. Three counts (0.1 mL aliquots each) were taken per replicate per time point. Each sample had a total volume of 150 mL. Fed cultures were fed on days 4 and 5.

Sample	Condition	Date	Time	Count 1	Count 2	Count3	Density (cells/mL)
Lm5-FedA	fed	2019-08-27	16:15:00	31	27	21	263.3
Lm5-FedB	fed	2019-08-27	16:15:00	26	29	19	246.7
Lm5-FedC	fed	2019-08-27	16:15:00	28	30	19	256.7
Lm5-FedA	fed	2019-08-28	14:15:00	22	23	23	226.7
Lm5-FedB	fed	2019-08-28	14:15:00	23	28	21	240
Lm5-FedC	fed	2019-08-28	14:15:00	27	25	20	240
Lm5-FedA	fed	2019-08-29	11:30:00	17	25	19	203.3
Lm5-FedB	fed	2019-08-29	11:30:00	20	21	23	213.3
Lm5-FedC	fed	2019-08-29	11:30:00	20	19	21	200
Lm5-FedA	fed	2019-08-30	10:50:00	23	22	18	210
Lm5-FedB	fed	2019-08-30	10:50:00	22	21	16	196.7
Lm5-FedC	fed	2019-08-30	10:50:00	29	34	30	310
Lm5-FedA	fed	2019-08-31	12:00:00	19	31	22	240
Lm5-FedB	fed	2019-08-31	12:00:00	29	24	32	283.3
Lm5-FedC	fed	2019-08-31	12:00:00	30	45	26	336.7
Lm5-StrA	starved	2019-08-27	16:15:00	29	21	24	246.7
Lm5-StrB	starved	2019-08-27	16:15:00	27	36	23	286.7
Lm5-StrC	starved	2019-08-27	16:15:00	24	21	26	236.7
Lm5-StrA	starved	2019-08-28	14:15:00	22	25	16	210
Lm5-StrB	starved	2019-08-28	14:15:00	14	24	33	236.7
Lm5-StrC	starved	2019-08-28	14:15:00	26	28	24	260
Lm5-StrA	starved	2019-08-29	11:30:00	20	27	19	220
Lm5-StrB	starved	2019-08-29	11:30:00	18	23	30	236.7
Lm5-StrC	starved	2019-08-29	11:30:00	25	24	31	266.7

Table S4. Published ciliate predicted proteomes (for MAC) or genome assemblies (for MIC) used for comparisons of genome completeness and functional annotations.

Species	Version	URL	Publication
Blepharisma stoltei ATCC 30299 MAC		https://bleph.ciliate.org/common/downloads/bleph/Bsto_ATCC_protein.fasta	(43)
Blepharisma stoltei ATCC 30299 MAC+IES		https://bleph.ciliate.org/common/downloads/bleph/bsto_atcc_mac_plus_ies.fa	(42)
Euplotes vannus MAC	Mar 2018	https://evan.ciliate.org/common/downloads/evan/Euplotes_vannus_Mar2018_proteins.fasta	(64)
Ichthyophthirius multifiliis MAC		https://ich.ciliate.org/common/downloads/ich/img1_0407.aa.fsa	(65)
Oxytricha trifallax MAC	022112	https://oxy.ciliate.org/common/downloads/oxy/Oxytricha_trifallax_022112_aa.fasta	(25)
Oxytricha trifallax MIC		https://oxy.ciliate.org/common/downloads/oxy/Oxytricha_trifallax_micronuclear_assembly.fasta	(47)
Paramecium caudatum 43c3d MAC	v2.0	https://paramecium.i2bc.paris-saclay.fr/files/Paramecium/caudatum/43c3d/annotations/caudatum_43c3d_assembly_v1/pcaudatum_43c3d_annotation_v2.0.protein.fa	(6)
Paramecium tetraurelia strain 51 MAC	v2.0	https://paramecium.i2bc.paris-saclay.fr/files/Paramecium/tetraurelia/51/annotations/ptetraurelia_mac_51/ptetraurelia_mac_51_annotation_v2.0.protein.fa	(6)
Paramecium tetraurelia strain 51 MAC+IES	v1.0	https://paramecium.i2bc.paris-saclay.fr/files/Paramecium/tetraurelia/51/sequences/ptetraurelia_mac_51_with_ies.fa	(6)
Perkinsus marinus ATCC 50983 (outgroup)	GCF_000006405	https://ftp.ncbi.nlm.nih.gov/genomes/all/GCF/000/006/405/GCF_000006405.1_JCVI_PMG_1.0/GCF_000006405.1_JCVI_PMG_1.0_protein.faa.gz	
Pseudocohnilembus persalinus MAC	GCA_001447515	https://ftp.ncbi.nlm.nih.gov/genomes/all/GCA/001/447/515/GCA_001447515.1_ASM144751v1/GCA_001447515.1_ASM144751v1_protein.faa.gz	(66)
Stentor coeruleus MAC	Nov 2017	https://stentor.ciliate.org/common/downloads/stentor/S_coeruleus_Nov2017_proteins.fasta	(67)
Stylonychia lemnae MAC	Nov 2017	https://stylo.ciliate.org/common/downloads/stylo/stylo_protein.fa	(46)

Tetrahymena borealis MAC	Oct 2012	https://tet.ciliate.org/common/downloads/tet/T_borealis_oct2012_proteins.fasta	(68)
Tetrahymena elliotii MAC	Oct 2012	https://tet.ciliate.org/common/downloads/tet/T_elliotti_oct2012_proteins.fasta	(68)
Tetrahymena malaccensis MAC	Oct 2012	https://tet.ciliate.org/common/downloads/tet/T_malaccensis_oct2012_proteins.fasta	(68)
Tetrahymena thermophila MAC	Mar 2020	https://tet.ciliate.org/common/downloads/tet/T_thermophila_mar2020-Protein%20fasta.fasta	(69)
Tetrahyemna thermophila MIC	2016	https://tet.ciliate.org/common/downloads/tet/2016_mic.genome.fasta	(70)

Table S5. Primary antibodies used in this study.

**Target**	**Supplier**	**Catalog no.**	**Type**	**IF dilution (1º)**	**IF dilution (2º)**	**WB dilution (1º)**	**WB dilution (2º)**
H3K9ac	Merck	06-942-S	rabbit polyclonal IgG	1:100	1:200	1:3000	1:5000
H3K9me3	Merck	07-442	rabbit polyclonal IgG	1:100	1:2000	1:3000	1:5000
H3K4me3	Abcam	ab8580	rabbit polyclonal IgG	1:100	1:200	1:5000	1:5000
Histone H3	Abcam	ab1791	rabbit polyclonal IgG	1:100	1:200	1:3000	1:5000
Histone H4	Abcam	ab177840	rabbit monoclonal IgG	1:100	1:200	1:500	1:2500
6mA	Synaptic Systems	202 003	rabbit polyclonal IgG	1:2000	1:200	-	-

SI References

1. A. Prjibelski, D. Antipov, D. Meleshko, A. Lapidus, A. Korobeynikov, Using SPAdes de novo assembler. *Curr. Protoc. Bioinformatics* **70**, e102 (2020).
2. H. R. Gruber-Vodicka, B. K. B. Seah, E. Pruesse, phyloFlash: Rapid Small-Subunit rRNA Profiling and Targeted Assembly from Metagenomes. *mSystems* **5** (2020).
3. C. Quast, *et al.*, The SILVA ribosomal RNA gene database project: improved data processing and web-based tools. *Nucleic Acids Res.* **41**, D590-6 (2013).
4. R. M. Waterhouse, *et al.*, BUSCO applications from quality assessments to gene prediction and phylogenomics. *Mol. Biol. Evol.* **35**, 543–548 (2018).
5. E. V. Kriventseva, *et al.*, OrthoDB v10: sampling the diversity of animal, plant, fungal, protist, bacterial and viral genomes for evolutionary and functional annotations of orthologs. *Nucleic Acids Res.* **47**, D807–D811 (2019).
6. O. Arnaiz, E. Meyer, L. Sperling, ParameciumDB 2019: integrating genomic data across the genus for functional and evolutionary biology. *Nucleic Acids Res.* **48**, D599–D605 (2020).
7. D. Mapleson, G. Garcia Accinelli, G. Kettleborough, J. Wright, B. J. Clavijo, KAT: a K-mer analysis toolkit to quality control NGS datasets and genome assemblies. *Bioinformatics* **33**, 574–576 (2017).
8. B. Bushnell, J. Rood, E. Singer, BBMerge - accurate paired shotgun read merging via overlap. *PLoS ONE* **12**, e0185056 (2017).
9. B. Bushnell, BBTools. *BBMap* (March 20, 2023).
10. M. G. Grabherr, *et al.*, Full-length transcriptome assembly from RNA-Seq data without a reference genome. *Nat. Biotechnol.* **29**, 644–652 (2011).
11. G. Pertea, M. Pertea, GFF utilities: gffread and gffcompare. *F1000Res.* **9** (2020).
12. R. Vera Alvarez, L. S. Pongor, L. Mariño-Ramírez, D. Landsman, TPMCalculator: one-step software to quantify mRNA abundance of genomic features. *Bioinformatics* **35**, 1960–1962 (2019).
13. E. P. Nawrocki, D. L. Kolbe, S. R. Eddy, Infernal 1.0: inference of RNA alignments. *Bioinformatics* **25**, 1335–1337 (2009).
14. I. Kalvari, *et al.*, Rfam 14: expanded coverage of metagenomic, viral and microRNA families. *Nucleic Acids Res.* **49**, D192–D200 (2021).
15. H. Li, Minimap2: pairwise alignment for nucleotide sequences. *Bioinformatics* **34**, 3094–3100 (2018).
16. B. K. B. Seah, C. Emmerich, A. Singh, E. C. Swart, Improved methods for bulk cultivation and fixation of *Loxodes* ciliates for fluorescence microscopy. *Protist* **173**, 125905 (2022).
17. Y. Wang, X. Chen, Y. Sheng, Y. Liu, S. Gao, N6-adenine DNA methylation is associated with the linker DNA of H2A.Z-containing well-positioned nucleosomes in Pol II-transcribed genes in *Tetrahymena*. *Nucleic Acids Res.* **45**, 11594–11606 (2017).
18. F. Guérin, *et al.*, Flow cytometry sorting of nuclei enables the first global characterization of *Paramecium* germline DNA and transposable elements. *BMC Genomics* **18**, 327 (2017).
19. A. L. Torres-Machorro, R. Hernández, A. M. Cevallos, I. López-Villaseñor, Ribosomal RNA genes in eukaryotic microorganisms: witnesses of phylogeny? *FEMS Microbiol. Rev.* **34**, 59–86 (2010).
20. J. L. Collier, *et al.*, The protist *Aurantiochytrium* has universal subtelomeric rDNAs and is a host for mirusviruses. *Curr. Biol.* **33**, 5199-5207.e4 (2023).

21. A. C. Mascarenhas Dos Santos, A. T. Julian, P. Liang, O. Juárez, J.-F. Pombert, Telomere-to-Telomere genome assemblies of human-infecting *Encephalitozoon* species. *BMC Genomics* **24**, 237 (2023).

22. T. Luttermann, *et al.*, Establishment of a near-contiguous genome sequence of the citric acid producing yeast *Yarrowia lipolytica* DSM 3286 with resolution of rDNA clusters and telomeres. *NAR Genom. Bioinform.* **3**, lqab085 (2021).

23. J. A. Upcroft, K. G. Krauer, P. Upcroft, Chromosome sequence maps of the *Giardia lamblia* assemblage A isolate WB. *Trends Parasitol.* **26**, 484–491 (2010).

24. J. G. Gibbons, A. T. Branco, S. Yu, B. Lemos, Ribosomal DNA copy number is coupled with gene expression variation and mitochondrial abundance in humans. *Nat. Commun.* **5**, 4850 (2014).

25. E. C. Swart, *et al.*, The *Oxytricha trifallax* macronuclear genome: a complex eukaryotic genome with 16,000 tiny chromosomes. *PLoS Biol.* **11**, e1001473 (2013).

26. M. C. Yao, A. R. Kimmel, M. A. Gorovsky, A small number of cistrons for ribosomal RNA in the germinal nucleus of a eukaryote, *Tetrahymena pyriformis*. *Proc Natl Acad Sci USA* **71**, 3082–3086 (1974).

27. N. N. Bobyleva, B. N. Kudrjavtsev, I. B. Raikov, Changes of the DNA content of differentiating and adult macronuclei of the ciliate *Loxodes magnus* (Karyorelictida). *J. Cell Sci.* **44**, 375–394 (1980).

28. C.-S. Chin, *et al.*, Phased diploid genome assembly with single-molecule real-time sequencing. *Nat. Methods* **13**, 1050–1054 (2016).

29. R. Vaser, I. Sović, N. Nagarajan, M. Šikić, Fast and accurate *de novo* genome assembly from long uncorrected reads. *Genome Res.* **27**, 737–746 (2017).

30. M. Stanke, S. Waack, Gene prediction with a hidden Markov model and a new intron submodel. *Bioinformatics* **19 Suppl 2**, ii215-25 (2003).

31. D. E. Vetter, Prediction of genes in genomes with ambiguous genetic codes. *Zenodo* (2022) https:/doi.org/10.5281/zenodo.7056821.

32. A. M. Zahler, Z. T. Neeb, A. Lin, S. Katzman, Mating of the stichotrichous ciliate *Oxytricha trifallax* induces production of a class of 27 nt small RNAs derived from the parental macronucleus. *PLoS ONE* **7**, e42371 (2012).

33. P. Y. Sandoval, E. C. Swart, M. Arambasic, M. Nowacki, Functional diversification of Dicer-like proteins and small RNAs required for genome sculpting. *Dev. Cell* **28**, 174–188 (2014).

34. W. Fang, X. Wang, J. R. Bracht, M. Nowacki, L. F. Landweber, Piwi-interacting RNAs protect DNA against loss during *Oxytricha* genome rearrangement. *Cell* **151**, 1243–1255 (2012).

35. U. E. Schoeberl, H. M. Kurth, T. Noto, K. Mochizuki, Biased transcription and selective degradation of small RNAs shape the pattern of DNA elimination in *Tetrahymena. Genes Dev.* **26**, 1729–1742 (2012).

36. K. Mochizuki, N. A. Fine, T. Fujisawa, M. A. Gorovsky, Analysis of a piwi-related gene implicates small RNAs in genome rearrangement in *Tetrahymena*. *Cell* **110**, 689–699 (2002).

37. S. Karunanithi, *et al.*, Exogenous RNAi mechanisms contribute to transcriptome adaptation by phased siRNA clusters in *Paramecium*. *Nucleic Acids Res.* **47**, 8036–8049 (2019).

38. Q. Carradec, *et al.*, Primary and secondary siRNA synthesis triggered by RNAs from food bacteria in the ciliate *Paramecium tetraurelia*. *Nucleic Acids Res.* **43**, 1818–1833 (2015).

39. S. R. Lee, K. Collins, Two classes of endogenous small RNAs in *Tetrahymena thermophila*. *Genes Dev.* **20**, 28–33 (2006).

40. D. P. Singh, *et al.*, Genome-defence small RNAs exapted for epigenetic mating-type inheritance. *Nature* **509**, 447–452 (2014).

41. B. P. Jain, S. Pandey, WD40 Repeat Proteins: Signalling Scaffold with Diverse Functions. *Protein J.* **37**, 391–406 (2018).

42. B. K. B. Seah, *et al.*, MITE infestation accommodated by genome editing in the germline genome of the ciliate *Blepharisma*. *Proc Natl Acad Sci USA* **120**, e2213985120 (2023).

43. M. Singh, *et al.*, Origins of genome-editing excisases as illuminated by the somatic genome of the ciliate *Blepharisma*. *Proc Natl Acad Sci USA* **120**, e2213887120 (2023).

44. A. Böhne, *et al.*, Zisupton--a novel superfamily of DNA transposable elements recently active in fish. *Mol. Biol. Evol.* **29**, 631–645 (2012).

45. W. Makałowski, V. Gotea, A. Pande, I. Makałowska, Transposable elements: classification, identification, and their use as a tool for comparative genomics. *Methods Mol. Biol.* **1910**, 177–207 (2019).

46. S. H. Aeschlimann, *et al.*, The draft assembly of the radically organized *Stylonychia lemnae* macronuclear genome. *Genome Biol. Evol.* **6**, 1707–1723 (2014).

47. X. Chen, *et al.*, The architecture of a scrambled genome reveals massive levels of genomic rearrangement during development. *Cell* **158**, 1187–1198 (2014).

48. K. Ross, *et al.*, Tncentral: a prokaryotic transposable element database and web portal for transposon analysis. *MBio* **12**, e0206021 (2021).

49. N. L. Craig, “Transposases and Integrases” in *ELS*, John Wiley & Sons, Ltd, Ed. (Wiley, 2001) https:/doi.org/10.1038/npg.els.0000593.

50. E. L. Greer, *et al.*, DNA Methylation on N6-Adenine in C. elegans. *Cell* **161**, 868–878 (2015).

51. Y. Wang, *et al.*, A distinct class of eukaryotic MT-A70 methyltransferases maintain symmetric DNA N6-adenine methylation at the ApT dinucleotides as an epigenetic mark associated with transcription. *Nucleic Acids Res.* **47**, 11771–11789 (2019).

52. L. Y. Beh, *et al.*, Identification of a DNA N6-Adenine Methyltransferase Complex and Its Impact on Chromatin Organization. *Cell* **177**, 1781-1796.e25 (2019).

53. J. Liu, *et al.*, A METTL3-METTL14 complex mediates mammalian nuclear RNA N6-adenosine methylation. *Nat. Chem. Biol.* **10**, 93–95 (2014).

54. G.-Z. Luo, *et al.*, N6-methyldeoxyadenosine directs nucleosome positioning in *Tetrahymena* DNA. *Genome Biol.* **19**, 200 (2018).

55. Y. Sheng, *et al.*, Semi-conservative transmission of DNA N^6 -adenine methylation in a unicellular eukaryote. *BioRxiv* (2023) https:/doi.org/10.1101/2023.02.15.468708.

56. Y. Sheng, B. Pan, F. Wei, Y. Wang, S. Gao, Case Study of the Response of N6-Methyladenine DNA Modification to Environmental Stressors in the Unicellular Eukaryote *Tetrahymena thermophila*. *mSphere* **6**, e0120820 (2021).

57. P. Fajkus, *et al.*, Evolution of plant telomerase RNAs: farther to the past, deeper to the roots. *Nucleic Acids Res.* **49**, 7680–7694 (2021).

58. M.-L. Pardue, P. G. DeBaryshe, Drosophila telomeres: A variation on the telomerase theme. *Fly (Austin)* **2**, 101–110 (2008).

59. X. X. Maurer-Alcalá, Y. Yan, O. A. Pilling, R. Knight, L. A. Katz, Twisted Tales: Insights into genome diversity of ciliates using single-cell ’omics. *Genome Biol. Evol.* **10**, 1927–1939 (2018).

60. I. B. Raikov, Primitive never-dividing macronuclei of some lower ciliates. *Int. Rev. Cytol.* **95**, 267–325 (1985).

61. D. Sellis, *et al.*, Massive colonization of protein-coding exons by selfish genetic elements in *Paramecium* germline genomes. *PLoS Biol.* **19**, e3001309 (2021).

62. X. X. Maurer-Alcalá, R. Knight, L. A. Katz, Exploration of the germline genome of the ciliate *Chilodonella uncinata* through single-cell omics (transcriptomics and genomics). *MBio* **9** (2018).

63. L. Huang, F. Ma, A. Chapman, S. Lu, X. S. Xie, Single-Cell Whole-Genome Amplification and Sequencing: Methodology and Applications. *Annu. Rev. Genomics Hum. Genet.* **16**, 79–102 (2015).

64. X. Chen, *et al.*, Genome analyses of the new model protist *Euplotes vannus* focusing on genome rearrangement and resistance to environmental stressors. *Mol. Ecol. Resour* **19**, 1292–1308 (2019).

65. R. S. Coyne, *et al.*, Comparative genomics of the pathogenic ciliate *Ichthyophthirius multifiliis*, its free-living relatives and a host species provide insights into adoption of a parasitic lifestyle and prospects for disease control. *Genome Biol.* **12**, R100 (2011).

66. J. Xiong, *et al.*, Genome of the facultative scuticociliatosis pathogen *Pseudocohnilembus persalinus* provides insight into its virulence through horizontal gene transfer. *Sci. Rep.* **5**, 15470 (2015).

67. M. M. Slabodnick, *et al.*, The macronuclear genome of *Stentor coeruleus* reveals tiny introns in a giant cell. *Curr. Biol.* **27**, 569–575 (2017).

68. N. A. Stover, R. S. Punia, M. S. Bowen, S. B. Dolins, T. G. Clark, *Tetrahymena* Genome Database Wiki: a community-maintained model organism database. *Database (Oxford)* **2012**, bas007 (2012).

69. Y. Sheng, *et al.*, The completed macronuclear genome of a model ciliate *Tetrahymena thermophila* and its application in genome scrambling and copy number analyses. *Sci. China Life Sci.* **63**, 1534–1542 (2020).

70. E. P. Hamilton, *et al.*, Structure of the germline genome of *Tetrahymena thermophila* and relationship to the massively rearranged somatic genome. *eLife* **5** (2016).
